# Global cancer burden attributable to tobacco smoking in 2022: a differential analysis by smoking status, sex, and development level

**DOI:** 10.1093/ije/dyag239

**Published:** 2026-11-06

**Authors:** Veer Shah, Darshi Shah, Karan Shah, Jeanne Wu, Daniele Bruno, Paolo Boffetta, Monireh Sadat Seyyedsalehi

**Affiliations:** Icahn School of Medicine at Mount Sinai, New York, NY, United States; Renaissance School of Medicine at Stony, Brook University, Stony Brook, NY, United States; Stony Brook Cancer Center, Stony Brook University, Stony Brook, NY, United States; The Johns Hopkins University School of Medicine, Baltimore, MD, United States; Division of General Surgery, Department of Surgery, Icahn School of Medicine at Mount Sinai, New York, NY, United States; Department of Medical and Surgical Sciences, University of Bologna, Bologna, Italy; Renaissance School of Medicine at Stony, Brook University, Stony Brook, NY, United States; Stony Brook Cancer Center, Stony Brook University, Stony Brook, NY, United States; Department of Medical and Surgical Sciences, University of Bologna, Bologna, Italy; Department of Medical and Surgical Sciences, University of Bologna, Bologna, Italy

**Keywords:** tobacco smoking, cancer, Population Attributable Fraction, incidence, mortality, current and former smokers, Human Development Index, global cancer burden

## Abstract

**Background:**

Tobacco smoking is a leading preventable cause of cancer. We estimated its attributable cancer burden by smoking status (current vs. former), further stratified by cancer type, sex, country, and Human Development Index (HDI).

**Methods:**

The Population Attributable Fraction (PAF) for cancer incidence and mortality due to tobacco were estimated across 175 countries by using 2000 Global Burden of Disease smoking prevalence and 2022 Global Cancer Observatory cancer data. Relative risks (RRs) were derived from large pooled and meta-analyses, and population data from the World Bank. Countries were categorized by HDI.

**Results:**

The global tobacco PAF was 17.2% for cancer incidence and 22.4% for mortality. Lung cancer had the highest RR for current smokers (8.96) and laryngeal cancer for former smokers (4.65). Males had a higher cancer incidence (26.1%) and mortality (32.1%) burden than females (8.0% and 10.2%, respectively). Cancers with the highest smoking PAF were laryngeal (70.1%), lung (66.9%), pharyngeal (63.5%), oral cavity (38.5%), and bladder (39.4%). PAF estimates for incidence were 5.4% in low-HDI (49 countries), 15.0% in medium-HDI [41], 19.3% in high-HDI [32], and 17.4% in very-high-HDI [32] countries; corresponding mortality estimates were 6.4% (low-HDI), 17.4% (medium-HDI), 26.0% (high-HDI), and 23.6% (very-high-HDI), respectively.

**Conclusion:**

Approximately one in six cancer cases and one in four cancer deaths globally are attributable to tobacco smoking. Despite declining smoking prevalence, former smokers account for a substantial burden long after cessation. Males and higher-HDI countries bear a greater burden.

Key Messages

- Research question: What proportion of the global cancer burden in 2022 was attributable to tobacco smoking, and how did this burden differ by smoking status, sex, cancer type, country, and Human Development Index (HDI)?
- What was found: Tobacco smoking accounted for approximately one in six cancer cases and one in four cancer deaths worldwide, with the greatest burden among men, in higher-HDI countries, and for lung, laryngeal, pharyngeal, oral cavity, and bladder cancers, while former smokers continued to contribute substantially.
- Why it matters: These findings support stronger smoking-cessation programmes, sex- and region-specific prevention strategies, and continued surveillance of the long-term cancer burden among former smokers.

## Introduction

Cancer is a leading cause of morbidity and mortality worldwide, with substantial regional and socio-economic variation [1, 2]. Identifying modifiable environmental and behavioural risk factors is critical for effective cancer prevention [3, 4]. Tobacco smoking is a major modifiable risk factor, with carcinogenic effects across multiple organ systems [5, 6]. The International Agency for Research on Cancer (IARC) classifies tobacco smoke as a human carcinogen linked to cancers across multiple organ systems [7].

To understand the extent of the impact of any carcinogen, it is important to analyse its contribution to the cancer burden. Historically, the Population Attributable Fraction (PAF) has been used to quantify the proportion of cancer cases and deaths preventable by eliminating a causal risk factor [8]. Given large geographic and socio-economic differences in tobacco use, understanding how these translate into cancer burden is crucial. Recent studies have quantified cancer burden attributable to tobacco smoking by age, sex, country, year, and socio-demographic index [9–11].Two prior studies by Pandeya *et al.* and Al-Zalabani *et al.* stratified attributable cancer incidence to tobacco smoke by sex, cancer type, and country [12, 13]. Other studies, by Safiri *et al.* and Li *et al.*, have stratified attributable mortality and smoking prevalence by region/country, year, age, sex, and cancer type [14, 15].

To our knowledge, no prior study has separated the contributions of current and former smoking to both cancer incidence and mortality while also stratifying by cancer type, country, sex, and socio-economic development. These differences clarify the evolving epidemic, capture the legacy effect of smoking, and inform prevention policy.

We provide a consistent, evidence-based assessment of the 2022 global cancer burden attributable to tobacco smoking, estimating the PAF of cancer incidence and mortality by smoking status (current vs. former), sex, Human Development Index (HDI), country, and cancer type [16].

## Methods

The prevalence of tobacco smoking in 2000 by sex and country was obtained from the Global Burden of Disease (GBD) study. The status of tobacco smoking was categorized as either ‘former’ or ‘current’. ‘Former’ smokers are defined as those who had quit the smoking of all tobacco products for ≥6 months [17–19], while ‘current’ smokers are individuals who continue to actively smoke tobacco. Smokeless tobacco and second-hand smoke were excluded. Smoking duration, cigarettes per day, and other aspects (e.g. [17] the smoking of cigars and pipes, industrial or non-industrial cigarettes, type of tobacco curing) were not considered. Because smoking is modifiable, the PAF estimates the cases that would not have occurred if exposure were eliminated [20].

Following the IARC, we included smoking-associated cancers of the oral cavity, nasopharynx, oropharynx, hypopharynx, larynx, oesophagus, stomach, colorectal, liver, sinonasal, lung, breast, cervix, mucinous ovary, kidney, bladder, and myeloid leukaemia [21].

The sex-specific numbers of incident cases and deaths from different cancer types for each country were derived from the 2022 Global Cancer Observatory (GLOBOCAN) database [1, 22]. Due to GLOBOCAN’s lack of data on subtypes of ovarian cancer, we estimated the subtype proportions by using the World Health Organization’s IARC Cancer Incidence in Five Continents Volume XII (CI5-XII) database [23]. Mucinous ovarian cancer was taken as 10% of ovarian cases [24, 25]. Similarly, we calculated the ratio of the age-standardized incidence of myeloid leukaemia (International Classification of Diseases, 10th Revision codes C92–94) to that of total leukaemia (C91–95) [23]. Myeloid leukaemia represented ∼45% of leukaemia cases [26, 27]. Of note, sinonasal cancer-incidence data were also obtained from the CI5-XII database [Nose, sinuses etc. (C30–31), age-standardized (world) incidence rates] and multiplied by the population of each country to calculate the total case numbers [23]. Sinonasal mortality was estimated by applying country-specific nasopharyngeal mortality-to-incidence ratios to sinonasal incidence.

The sex-specific prevalences of the rates for current and former smoking for 175 countries were derived from the GBD study [10]. An adequate latency between exposure and malignancy is required; for smoking-related cancers, this is estimated at ∼20 years [28, 29]. Thus, smoking rates from the year 2000 were extracted for all countries. This 20-year lag was applied uniformly to both current and former smokers: the year-2000 prevalence of current smoking and of former smoking was treated as the exposure relevant to cancers occurring in 2022. This interval approximates the median latency between carcinogenic exposure and the clinical appearance of the cancers considered. For former smokers, risk was therefore assigned on the basis of former-smoking prevalence in 2000; because the excess risk conferred by smoking declines only gradually over the one to two decades following cessation, individuals who were former smokers in 2000 still carried elevated risk relevant to cancers diagnosed in 2022.

Incidence and mortality data were unavailable for 18 countries across the HDI categories, including Hong Kong and Taiwan. The population of each country was derived from the 2022/23 database of the World Bank [30]. The relative risks (RRs) for tobacco-associated cancers were derived from a systematic review of large-scale pooled analyses and meta-analyses [31–34]. Separate RRs were obtained for former- and current-smoking status. Studies were identified by searching ‘meta-analysis’ and ‘[cancer type]’ (English only). The reference meta-analysis reported separate analyses for sinonasal and nasopharyngeal cancers among current users only [31]. For former users, these cancer types were analysed as a combined category. The same RR estimates were applied to both cancer incidence and mortality, as the pooled analyses and meta-analyses used did not provide separate incidence- and mortality-specific risk estimates for most cancer sites.

Expressed as a percentage, the PAF represents the proportion of cancer cases in the total population attributed to tobacco smoking. The HDI is a composite index measuring the average achievement of a country in key dimensions of health, education, and standard of living [35]. The PAFs were computed by using the following formulae derived from Levin [36]:


$${\mathrm{PAF}}_{c}\;=\;\frac{P_{c}\;\times \;({RR}_{c}\;-\;1)}{P_{c}\;\times \;({RR}_{c}\;-\;1)\;+\;P_{f}\;\times \;({RR}_{f}\;-\;1)\;+\;1}$$

$${\mathrm{PAF}}_{f}\;=\;\frac{P_{f}\;\times \;({RR}_{f}\;-\;1)}{P_{f}\;\times \;({RR}_{f}\;-\;1)\;+\;P_{c}\;\times \;({RR}_{c}\;-\;1)\;+\;1}$$

where *RR*_c_ represents the relative risk of developing the disease among current smokers compared with never smokers; *RR*_f_ represents the relative risk among former smokers compared with never smokers; *P*_c_ represents the prevalence of current smokers; and *P*_f_ represents the prevalence of former smokers.

We employed the Levin PAF because it requires only summary estimates of exposure prevalence and RRs, which were the data consistently available across all 175 countries, and because it permits transparent and reproducible comparison across countries, sexes, smoking statuses, and cancer types. More data-intensive approaches, such as the smoking impact ratio (SIR), which uses lung cancer mortality as an indirect marker of accumulated population smoking exposure, or dose–response models incorporating smoking duration and intensity, were not feasible because country-specific data on smoking duration, cigarettes smoked per day, and the distribution of time since cessation were not uniformly available across the countries studied. To partially capture exposure heterogeneity within the Levin framework, current and former smokers were modelled separately, with each assigned status-specific RRs.

### Calculating attributable cases

The total number of tobacco-attributable cases or deaths attributed to current smoking (*N*_c_) or former smoking (*N*_f_) is calculated by using the following formulae:


$$\;N_{c}=\;({\mathrm{PAF}}_{c}\;\times \;N)$$

$$N_{f}\;=\;({\mathrm{PAF}}_{f}\;\times \;N)$$

$$N_{T}\;=\;N_{c}\;+\;N_{f}$$

where *PAF*_c_ denotes the PAF for current smoking; *N*_c_ denotes the total number of cases or deaths attributable to current smokers; *PAF*_f_ denotes the PAF for former smoking; *N*_f_ denotes the total number of cases or deaths attributable to former smokers; and *N*_T_ denotes the total number of tobacco-attributable cases or deaths. In these formulae, *N* represents the total number of incident cases (for incidence estimates) or deaths (for mortality estimates) of the cancer of interest in the population under consideration.

The country-level total PAF was calculated as:


$$\mathrm{Total}\;\mathrm{Incidence}\;\mathrm{PAF}=\frac{\mathrm{Attributable}\;\mathrm{Incident}\;\mathrm{Cases}\;(\mathrm{including}\;\mathrm{current}\;\mathrm{and}\;\mathrm{former})}{\mathrm{Incident}\;\mathrm{cases}\;of\;\mathrm{all}\;\mathrm{cancers}\;(\mathrm{including}\;\mathrm{non}-\mathrm{tobacco}-\mathrm{related}\;\mathrm{cancers})}$$

$$\mathrm{Total}\;\mathrm{Mortality}\;\mathrm{PAF}=\frac{\mathrm{Attributable}\;\mathrm{Mortality}\;\mathrm{Cases}\;(\mathrm{including}\;\mathrm{current}\;\mathrm{and}\;\mathrm{former})}{\mathrm{Deaths}\;\mathrm{from}\;\mathrm{all}\;\mathrm{cancers}\;(\mathrm{including}\;\mathrm{non}-\mathrm{tobacco}-\mathrm{related}\;\mathrm{cancers})}$$

The global PAF was calculated as:


$$\mathrm{Global}\;\mathrm{Incidence}\;\mathrm{PAF}=\frac{\;\mathrm{Worldwide}\;\mathrm{Attributable}\;\mathrm{Incident}\;\mathrm{Cases}\;(\mathrm{including}\;\mathrm{current}\;\mathrm{and}\;\mathrm{former})}{\mathrm{Incident}\;\mathrm{cases}\;of\;\mathrm{all}\;\mathrm{cancers}\;in\;\mathrm{all}\;\mathrm{countries}\;(\mathrm{including}\;\mathrm{non}-\mathrm{tobacco}-\mathrm{related}\;\mathrm{cancers})}$$

$$\mathrm{Global}\;\mathrm{Mortality}\;\mathrm{PAF}=\frac{\mathrm{Worldwide}\;\mathrm{Attributable}\;\mathrm{Mortality}\;\mathrm{Cases}\;(\mathrm{including}\;\mathrm{current}\;\mathrm{and}\;\mathrm{former})}{\mathrm{Deaths}\;\mathrm{from}\;\mathrm{all}\;\mathrm{cancers}\;in\;\mathrm{all}\;\mathrm{countries}\;(\mathrm{including}\;\mathrm{non}-\mathrm{tobacco}-\mathrm{related}\;\mathrm{cancers})}$$

The same structures were applied within subgroups (sex, HDI, and smoking status).

## Results

Globally in 2000, there were an estimated 1.8 billion current smokers (1.4 billion male, 0.4 billion female) and 0.8 billion former smokers (0.6 billion male, 0.2 billion female). The smoking prevalence of current smokers exceeded 20% in 147 countries for males and in 38 countries for females. Similarly, the smoking prevalence of former smokers exceeded 20% in 99 countries for males and in 12 countries for females (Supplementary Table 1).

Lung cancer had the highest RR in current smokers (8.96) and laryngeal cancer had the highest in former smokers (4.65); lung cancer in former smokers had the second-highest RR (3.85) (Table 1). Across other sites, current-smoker RRs ranged from 1.14 (colorectal) to 6.76 (pharyngeal) and former-smoker RRs ranged from 1.17 to 2.28.

**Table 1:** Relative risk of cancer associated with current and former smoking, by cancer type.

Cancer type	RR (95% CI)	Source of RR
**Current smoking**		
Bladder	2.77 (2.17–3.54)	Gandini *et al.*, 2008
Breast	1.11 (1.06–1.16)	Macacu *et al.*, 2015
Cervix	1.83 (1.51–2.21)	Gandini *et al.*, 2008
Colorectal	1.14 (1.10–1.18)	Botteri *et al.*, 2020
Oesophagus	2.5 (2.00–3.13)	Gandini *et al.*, 2008
Kidney	1.52 (1.33–1.74)	Gandini *et al.*, 2008
Larynx	6.98 (3.14–15.5)	Gandini *et al.*, 2008
Liver	1.56 (1.29–1.87)	Gandini *et al.*, 2008
Lung	8.96 (6.73–12.1)	Gandini *et al.*, 2008
Mucinous ovary	1.78 (1.52–2.07)	Santucci *et al.*, 2019
Myeloid leukaemia^a^	1.09 (0.7–1.7)	Gandini *et al.*, 2008
Nasopharynx	2.23 (1.13–4.42)	Gandini *et al.*, 2008^b^
Oral cavity	3.43 (2.37–4.94)	Gandini *et al.*, 2008^b^
Pancreatic	1.7 (1.51–1.91)	Gandini *et al.*, 2008
Pharynx	6.76 (2.86–16.0)	Gandini *et al.*, 2008
Sinonasal^a^	1.49 (0.59–3.96)	Gandini *et al.*, 2008
Stomach	1.64 (1.37–1.95)	Gandini *et al.*, 2008
**Former smoking**		
Bladder	1.72 (1.46–2.04)	Gandini *et al.*, 2008
Breast	1.09 (1.06–1.12)	Macacu *et al.*, 2015
Cervix	1.26 (1.11–1.42)	Gandini *et al.*, 2008
Colorectal	1.17 (1.15–1.20)	Botteri *et al.*, 2020
Oesophagus	2.03 (1.77–2.33)	Gandini *et al.*, 2008
Kidney	1.25 (1.14–1.37)	Gandini *et al.*, 2008
Larynx	4.65 (3.35–6.45)	Gandini *et al.*, 2008
Liver	1.49 (1.06–2.10)	Gandini *et al.*, 2008
Lung	3.85 (2.77–5.34)	Gandini *et al.*, 2008
Mucinous ovary^a^	1.07 (0.98–1.18)	Santucci *et al.*, 2019
Myeloid leukaemia^a^	1.27 (0.28–5.83)	Gandini *et al.*, 2008
Nasopharynx	1.39 (1.08–1.79)	Gandini *et al.*, 2008
Oral cavity^a^	1.4 (0.99–2.00)	Gandini *et al.*, 2008
Pancreatic	1.18 (1.04–1.33)	Gandini *et al.*, 2008
Pharynx^a^	2.28 (0.95–5.50)	Gandini *et al.*, 2008
Sinonasal	1.39 (1.08–1.79)	Gandini *et al.*, 2008
Stomach	1.31 (1.17–1.46)	Gandini *et al.*, 2008

The same RRs were applied to both cancer incidence and mortality, as the pooled analyses and meta-analyses used did not report separate incidence- and mortality-specific estimates for most cancer sites.

a The 95% CI includes values below 1.

b The reference reported sinonasal and nasopharyngeal cancers separately only for current smokers (nasal-sinus cavity RR 1.49; 95% CI 0.59–3.96, three studies, *I*^2^ = 0%; nasopharynx RR 2.23; 95% CI 1.13–4.42, seven studies, *I*^2^ = 77%; test for difference between sites *P* = .36). No separate analysis was conducted for former smokers, which explains the identical RRs for these cancer types among former smokers.

CI, confidence interval.

These risk estimates translated into substantial PAFs. The cancers with the highest smoking-attributable burden were lung (66.9% overall PAF), laryngeal (70.1%), pharyngeal (63.5%), oral cavity (38.5%), and bladder (39.4%) cancers. This burden corresponded to 1 600 000 attributable lung cancer cases and 1 200 000 attributable lung cancer deaths, with additional substantial burdens for laryngeal (130 000 cases, 71 000 deaths), pharyngeal (121 000 cases, 58 000 deaths), oral cavity (150 000 cases and 72 000 deaths), and bladder cancer (221 000 cases, 78 000 deaths) (Table 2).

**Table 2:** Cancer cases and deaths attributable to tobacco smoking (current and former) and overall attributable fraction of cancer incidence, by cancer type and sex, 2022.

Cancer	Sex	Incident cases attributable to smoking (current + former)	Deaths attributable to smoking (current + former)	Overall attributable fraction of cancer incidence, both sexes (%)
Bladder	Male	206 980	72 930	39.4
	Female	34 820	12 120	
Breast	Female	50 490	12 350	2.2
Cervix	Female	45 650	23 340	6.9
Colorectal	Male	86 740	40 140	6.0
	Female	29 380	13 280	
Oesophagus	Male	157 450	136 820	34.6
	Female	18 940	16 290	
Kidney	Male	54 090	19 420	15.7
	Female	14 140	4670	
Larynx	Male	122 030	66 230	70.1
	Female	10 260	5080	
Liver	Male	141 030	122 310	18.4
	Female	18 090	15 930	
Lung	Male	1 236 090	969 510	66.9
	Female	422 610	277 950	
Mucinous ovary	Female	2520	1630	7.8
Myeloid leukaemia	Male	9950	6000	6.1
	Female	3310	1870	
Nasopharynx	Male	31 930	20 030	28.9
	Female	2770	1540	
Oral cavity	Male	126 570	62 040	38.5
	Female	23 370	9490	
Pancreatic	Male	61 800	56 840	16.9
	Female	24 170	22 090	
Pharynx	Male	109 320	53 770	63.5
	Female	12 860	5700	
Sinonasal	Male	2390	1420	15.2
	Female	500	260	
Stomach	Male	149 200	100 500	17.9
	Female	24 250	15 850	

Current-smoking and former-smoking cases were rounded to the nearest multiple of 10. Overall cancer-incidence burdens were rounded to the nearest multiple of 1000.

Cancer PAFs demonstrated a strong positive correlation with HDI levels (Fig. 1)—a pattern that was consistent across the most burdensome cancers. For lung cancer in male current smokers, the PAFs were 59.0% in very-high-HDI countries, 69.8% in high-HDI countries, 67.2% in medium-HDI countries, and 48.9% in low-HDI countries. Despite slightly lower PAFs, the absolute burden was concentrated in higher-HDI settings: 480 000 lung cancer deaths in high-HDI countries compared with 241 000, 110 000, and 4600 in very-high-HDI, medium-HDI, and low-HDI countries, respectively (Supplementary Table 4). Among male former smokers, the attributable lung cancer deaths totalled 74 000 in very-high-HDI countries, 44 000 in high-HDI countries, 15 000 in medium-HDI countries, and 1300 in low-HDI countries (Supplementary Table 6). Figure 2a and b demonstrates the PAF percentages for male and female smokers across various HDI categories. For the number of incidence cases in both sexes stratified by cancer type and HDI categories, in current smokers refer to Supplementary Table 3 and in former smokers refer to Supplementary Table 5.

**Figure 1:**
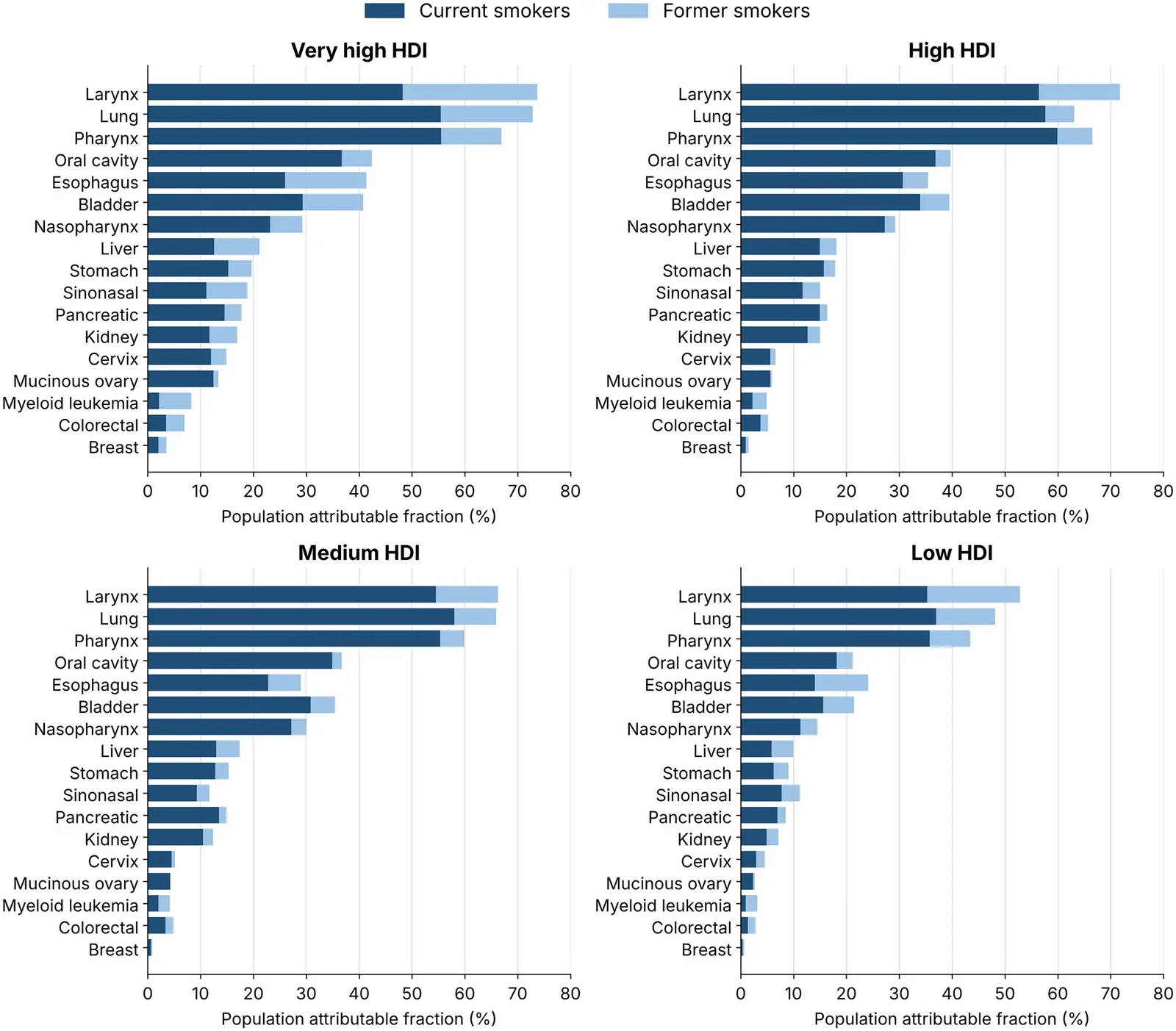
PAF of cancer incidence due to tobacco smoking by cancer type and HDI category, both sexes, 2022. Horizontal stacked bars show the PAF (%) of incident cancer cases due to current smoking (dark blue) and former smoking (light blue) for each smoking-related cancer, in countries grouped by HDI category (very high, high, medium, and low). Cancers are ordered by total PAF in very-high-HDI countries.

**Figure 2:**
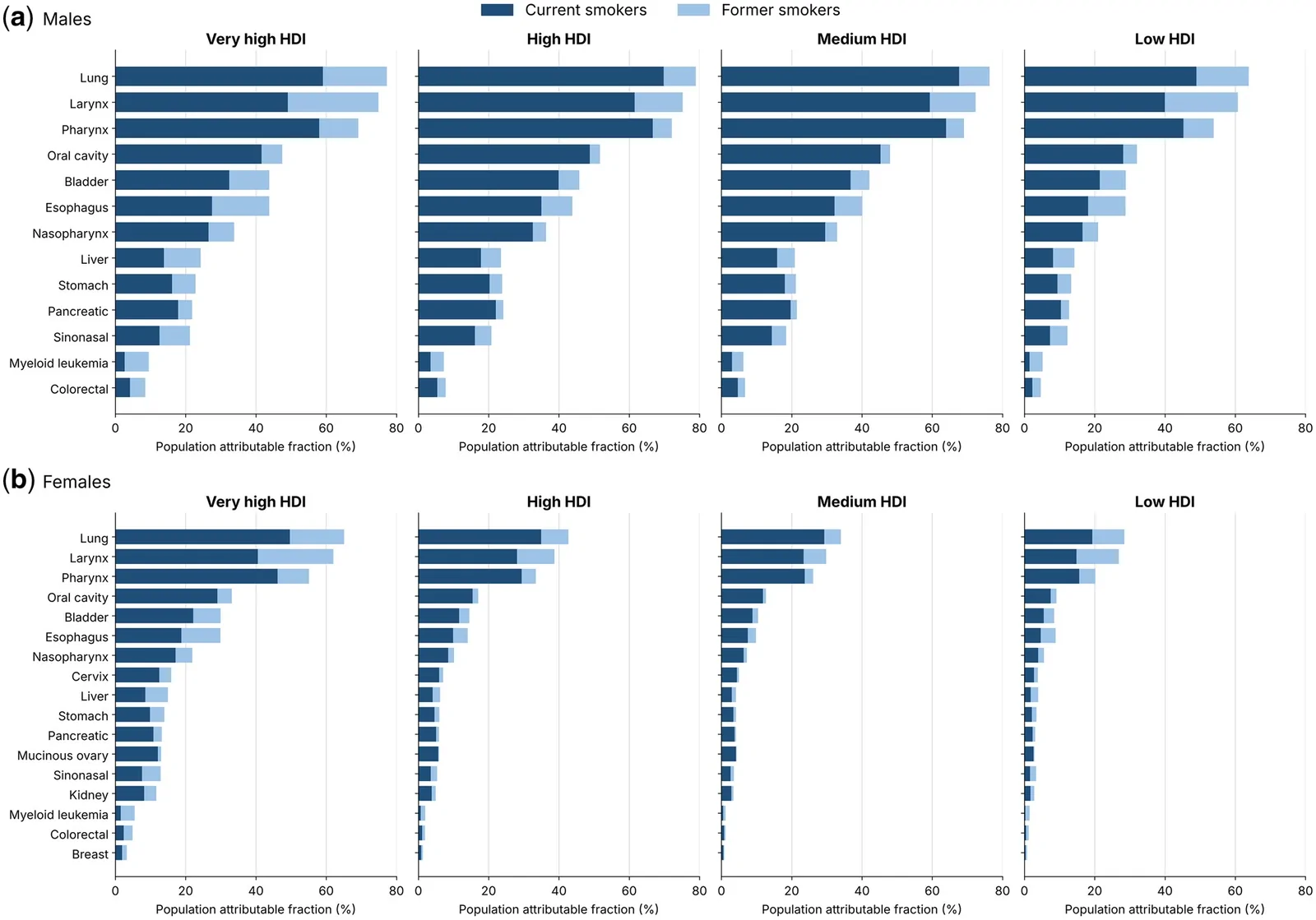
PAF of cancer incidence due to tobacco smoking by cancer type and HDI category in (A) males and (b) females, 2022. Horizontal stacked bars show the PAF (%) of incident cancer cases due to current smoking (dark blue) and former smoking (light blue), by HDI category. The same horizontal scale is used in both panels so that the sexes can be compared directly. Breast, cervical, and mucinous ovarian cancers are shown for females only.

In very-high-HDI countries such as Sweden, New Zealand, and the UK, former smokers outnumber current smokers (e.g. UK: 45% vs. 26% of males), narrowing the gap between current- and former-smoker PAFs (UK male lung cancer: 47.1% vs. 29.9%). In lower-HDI countries with weaker cessation, the gap is far larger (e.g. Cambodia: 77.3% vs. 5.2%).

Figure 3a and b maps the incidence PAFs by country. Figure 4a and b maps the mortality PAFs by country.

**Figure 3:**
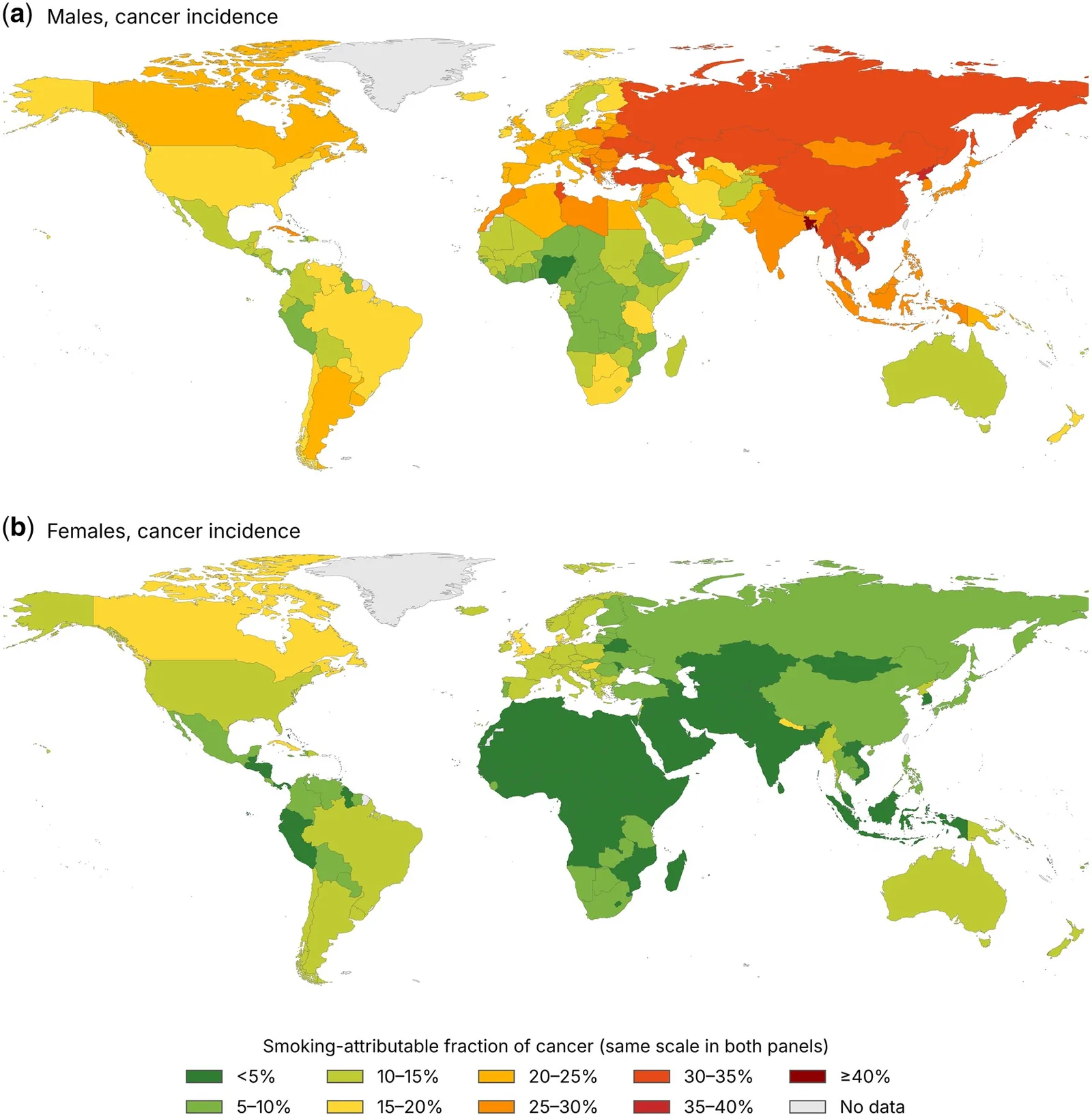
PAF of cancer incidence due to tobacco smoking by country in (A) males and (B) females, 2022. Countries are shaded by the PAF, i.e. the proportion of all incident cancer cases attributable to tobacco smoking, in nine categories of 5 percentage points from <5% to ≥40%. The same scale is used in both panels. Countries without data are shown in grey. Country boundaries: Natural Earth (public domain).

**Figure 4:**
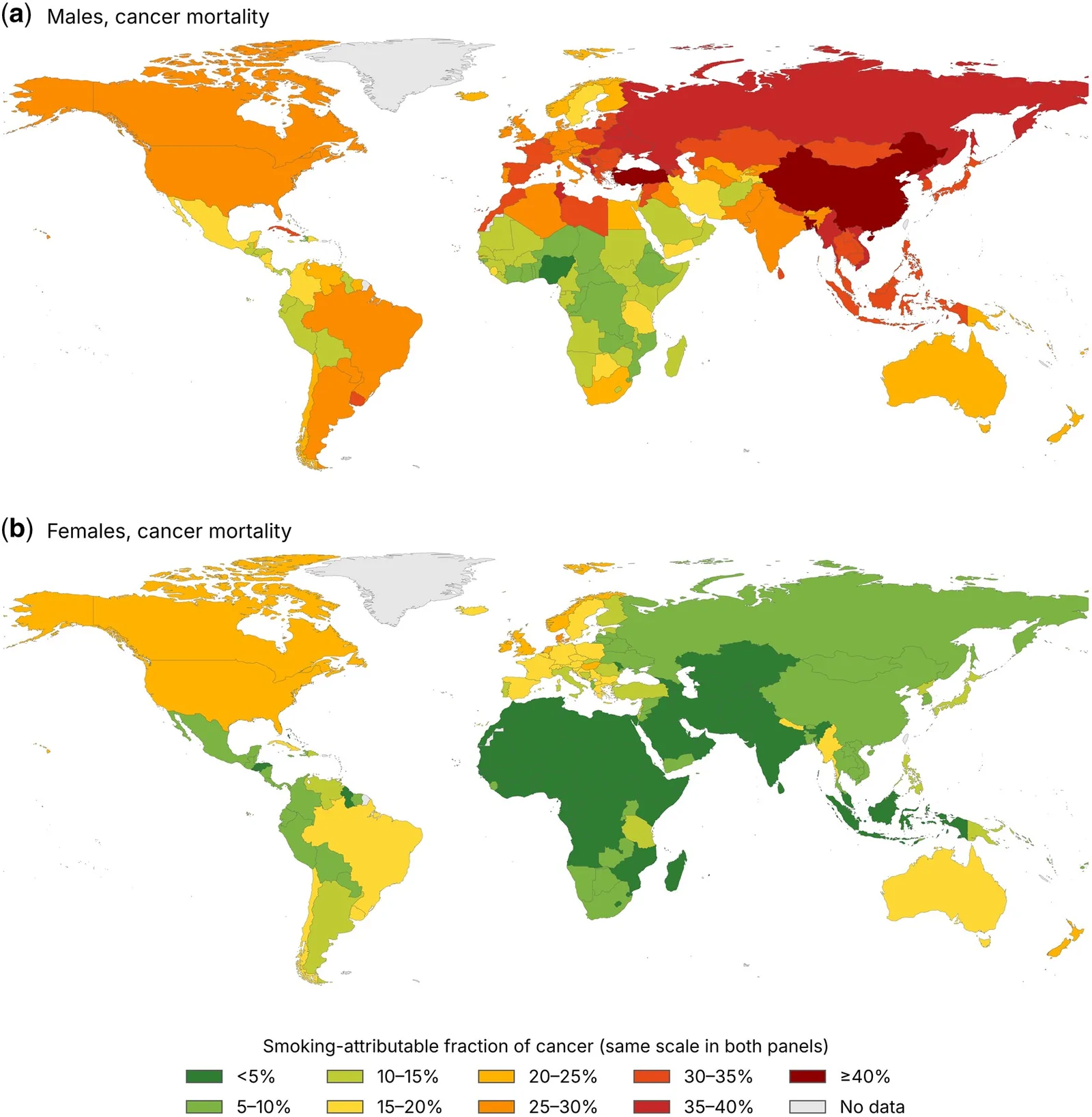
PAF of cancer mortality due to tobacco smoking by country in (A) males and (B) females, 2022. Countries are shaded by the PAF, i.e. the proportion of all cancer deaths attributable to tobacco smoking, in nine categories of 5 percentage points from <5% to ≥40%. The same scale is used in both panels. Countries without data are shown in grey. Country boundaries: Natural Earth (public domain).

At the country level, the highest overall PAFs for incidence across all smoking-attributable cancers were observed in Bangladesh (24.7%), North Korea (23.6%), Cuba (23.0%), Myanmar (22.8%), and Serbia (22.4%) (Supplementary Figure 1A and Supplementary Table 7), while the mortality PAFs across all smoking-attributable cancers were highest in Turkey (31.1%), Hungary (30.0%), China (29.0%), Serbia (28.1%), and Montenegro (27.6%) (Supplementary Figure 1B and Supplementary Table 7). Geographic visualization of these patterns is presented by sex for both incidence (Fig. 3a and b) and mortality (Fig. 4a and b).

A consistent and striking pattern emerged across all analyses: across gender-neutral solid tumours, males consistently bore a disproportionately higher tobacco-attributable cancer burden than did females. Overall, males had a 26.1% incidence burden compared with 8.0% in females and a 32.1% mortality burden versus 10.2% in females. This male predominance was evident across individual cancer sites, with lung and laryngeal PAFs of 78.7% vs. 46.6% and 73.8% vs. 43.9%, respectively (Table 2).

The sex disparity persisted across all HDI levels and smoking statuses. For pharyngeal cancer, the current-smoker male PAFs exceeded the female PAFs at every development level (e.g. 66.7% vs. 29.4% in high-HDI countries) and the same male excess held among former smokers.

## Discussion

This study offers a detailed analysis of the global cancer burden attributable to tobacco smoking, with its novelty lying in emphasizing disparities across former and current smokers, categorized by cancer type, HDI groups, countries, and sex.

Current smokers accounted for most of the burden, with higher PAFs and RRs across nearly all sites, especially lung and larynx. In many high-HDI countries (e.g. Sweden, the UK, New Zealand), former smokers now outnumber current smokers, narrowing the PAF gap and underscoring the lasting impact of past smoking.

This disparity was narrower in high-cessation, very-high-HDI countries and far wider in low-HDI and medium-HDI countries. Countries with a higher proportion of former smokers, such as the UK, had major cultural shifts after the landmark studies by Doll and Hill [37, 38] linked tobacco smoking to lung cancer. Sweden reduced smoking partly through smokeless tobacco (snus) [39]. Nevertheless, current smokers still outnumbered former smokers in most countries, underscoring the ongoing need for cessation efforts.

The highest PAFs were in respiratory and aerodigestive cancers, in order: larynx, lung, pharynx, oral cavity, and bladder [29, 40–44]. However, lung cancer had the greatest absolute burden. The RRs were highest for lung cancer in current smokers and for laryngeal cancer in former smokers. This difference reflects the higher baseline incidence of lung cancer and shows that prevention must weigh both tobacco association and disease frequency.

Higher-HDI countries show elevated PAFs despite stronger tobacco control, reflecting the legacy of mid-twentieth-century smoking epidemics (e.g. 480 000 lung cancer deaths in high-HDI countries vs. 4600 in low-HDI countries); former smokers remain at risk, sustaining the burden. In low-HDI countries such as Syria and Rwanda, current smokers dominate [45, 46]. Very-high-HDI countries such as Germany show a more balanced burden between current and former smokers, reflecting successful cessation supported by strong policies [47, 48].

Consistently with prior studies, men smoked more than women across all HDI groups [9, 49]. The higher male PAFs for gender-neutral cancers such as pharynx call for strategies targeting tobacco use in men.

Our study contributes to existing literature through comprehensive analyses of the global tobacco-related cancer burden, especially by differentiating impacts on former and current smokers along with risk stratifications by cancer type, sex, HDI, and country. By leveraging the most recent GLOBOCAN 2022 data, our study builds upon prior research such as the 2019 GBD with updated, more detailed coverage of diverse and expanded cancer types as well as refined RR estimates [10]. Our inclusion of a broad spectrum of IARC-recognized cancer types associated with tobacco, including those less frequently analysed, such as sinonasal, mucinous ovarian, and myeloid leukaemia, ensures a more comprehensive evaluation of the carcinogenic burden of smoking tobacco. Methodologically, this study integrates multiple high-quality peer-reviewed primary sources, resulting in precise PAF calculations. In addition, a key strength of our methodology was the deliberate selection of the year 2000 as the baseline for smoking-prevalence data, allowing a sufficient latency period (20+ years) for known cancer-development timelines following exposure to carcinogens present in tobacco, strengthening the reliability of time-dependent risk assessments.

Using the comparative risk-assessment framework of GBD 2019, Safiri *et al.* estimated that ∼2.5 million cancer deaths were attributable to tobacco smoking worldwide, corresponding to almost one in four cancer deaths—a figure closely consistent with our global mortality PAF of 22.4% [10, 15]. Both identify a greater burden among men and a positive gradient with development, with high burdens in high-income North America and Western and Central Europe paralleling our elevated high-HDI PAFs. Differences between the estimates stem largely from methodological choices: the GBD applies age-specific RRs and a summary exposure value derived from the SIR within a comparative risk assessment, whereas we applied the Levin PAF using point-prevalence estimates and pooled RRs. Our analysis also uses GLOBOCAN 2022, separates current from former smokers, and includes cancers (e.g. sinonasal, mucinous ovarian, myeloid leukaemia) that the GBD does not always report separately; these and differing exposure definitions likely explain the modest discrepancies.

This study has limitations. Variations in national registry data prevented accounting for duration and pack-years of tobacco use, limiting dose- and duration-based stratification. Future studies quantifying these effects in former and current smokers will improve understanding of the impact of smoking across cancer types. We did not use region-specific RRs, which may have introduced bias due to differences in tobacco products, smoking patterns, and genetics. Second-hand and smokeless-tobacco exposures were excluded, likely underestimating the total burden. Reliance on point-prevalence data and uniform 20-year latency may not have captured variation across cancers or populations. Self-reported smoking data may also have underestimated use in groups with low social acceptability. Smoking-prevalence and cancer data presented additional challenges due to limited national surveillance in many low- and middle-income countries, for which estimates are often extrapolated from neighbouring regions, assuming similar risk profiles and cancer patterns. This may have introduced systematic error. Additionally, GLOBOCAN’s reliance on variable registry quality and modelled estimates affects the precision of PAF calculations. Several RR estimates were drawn from an earlier comprehensive meta-analysis (Gandini *et al.*); although more recent site-specific meta-analyses were incorporated where available (for colorectal, breast, and ovarian cancers), secular changes in cigarette composition and smoking behaviour may not have been fully reflected. Because the same RRs were applied to both incidence and mortality, our estimates assume that smoking influences cancer occurrence and case fatality to a similar relative degree; if smoking additionally affects survival or stage at diagnosis, then mortality PAFs may be biased relative to incidence PAFs. We used the Levin PAF rather than the SIR or dose–response models; this maximizes comparability, but may not have captured the intensity- and duration-dependence of risk, potentially under- or overestimating the burden in some settings. Finally, we did not adjust for age: smoking-related RRs vary with age, age structures differ across countries, and initiation and cessation patterns have shifted across birth cohorts. Combining non-age-specific RRs with single-time-point prevalence incompletely captures these factors and may limit cross-country comparability.

Future work should incorporate smokeless tobacco and e-cigarettes, use longitudinal designs to assess the long-term effects of tobacco control, and improve country-specific data, notably for Afghanistan.

## Ethics approval

This study used publicly available, aggregated, and anonymized data (GLOBOCAN 2022, the Global Burden of Disease study, and the World Bank) and did not involve human participants or identifiable personal information; ethics approval and informed consent were therefore not required.

## Supplementary Material

dyag239_Supplementary_Data

## Data Availability

The data underlying this article are publicly available: cancer-incidence and mortality estimates from GLOBOCAN 2022 (Global Cancer Observatory, https://gco.iarc.fr); tobacco-smoking prevalence from the Global Burden of Disease study (Institute for Health Metrics and Evaluation, https://ghdx.healthdata.org); and population data from the World Bank (https://data.worldbank.org).
