# Supplementary material for: Global cancer burden attributable to tobacco smoking in 2022: a differential analysis by smoking status, sex, and development level
Source: Int J Epidemiol. 2026 Nov 6;55(6):dyag239. doi: 10.1093/ije/dyag239 (PMC13743343; doi:10.1093/ije/dyag239)
Supplement: Supplementary file 1 [file dyag239_supplementary_data.docx]

**Global Cancer Burden Attributable to Tobacco Smoking in 2022: A Differential Analysis by Smoking Status, Sex, and Development Level**

*Veer Shah^1^, Darshi Shah^2,3^, Karan Shah^4^, Jeanne Wu^5^, Daniele Bruno^6^, Paolo Boffetta^2,3,6^, Monireh Sadat Seyyedsalehi^6*^*

^1^Icahn School of Medicine at Mount Sinai, New York, NY 10029, USA

^2^Renaissance School of Medicine at Stony Brook University, Stony Brook, NY, USA

^3^Stony Brook Cancer Center, Stony Brook University, Stony Brook, NY, USA

^4^The Johns Hopkins University School of Medicine, Baltimore, MD 21205, USA

^5^Division of General Surgery, Department of Surgery, Icahn School of Medicine at Mount Sinai, New York, NY 10029, USA

^6^Department of Medical and Surgical Sciences, University of Bologna, 40138 Bologna, Italy

*Corresponding author: Monireh Sadat Seyyedsalehi, Department of Medical and Surgical Sciences, University of Bologna, Via Massarenti 9, 40138 Bologna, Italy. Email: monireh.seyydsalehi@unibo.it

# **SUPPLEMENTARY MATERIAL**

**Contents**

Figure S1A.	Heat Map of Overall Incidence PAFs	3

Figure S1B.	Heat Map of Overall Mortality PAFs	4

Table S1.	Countries categorized by World Bank HDI and the Smoking Prevalence in each country for current and former smokers	4

Table S2.	Countries categorized by World Bank HDI and the PAF for incidence and mortality in each country for current and former smokers	13

Table S3.	Number of cancer incidence cases attributable to tobacco smoking in current smokers characterized by global, very high, high, medium, and low HDI categories (rounded to nearest whole number)	19

Table S4.	Number of cancer mortality cases attributable to tobacco smoking in current smokers characterized by global, very high, high, medium, and low HDI categories (rounded to nearest whole number)	20

Table S5.	Number of cancer incidence cases attributable to tobacco smoking in former smokers characterized by global, very high, high, medium, and low HDI categories (rounded to nearest whole number)	21

Table S6.	Number of cancer mortality cases attributable to tobacco smoking in former smokers characterized by global, very high, high, medium, and low HDI categories (rounded to nearest whole number)	22

Table S7.	Total cancer cases and PAF including all cancer types related to tobacco smoking for incidence and mortality by country	25

**Abbreviations:** PAF, population attributable fraction; RR, relative risk; CI, confidence interval; HDI, Human Development Index; GBD, Global Burden of Disease; GLOBOCAN, Global Cancer Observatory database; UNDP, United Nations Development Programme. These abbreviations apply throughout the supplementary tables and figures below.

**Figure S1A.** Heat Map of Overall Incidence PAFs

**Figure S1B.** Heat Map of Overall Mortality PAFs

| **Table S1.** Countries categorized by World Bank HDI and the Smoking Prevalence in each country for current and former smokers. | | | | |
| --- | --- | --- | --- | --- |
| **Very High HDI** | | | | |
| **Countries** | **Smoking Prevalence of Current Male Smokers (%)** | **Smoking Prevalence of Current Female Smokers (%)** | **Smoking Prevalence of Former Male Smokers (%)** | **Smoking Prevalence of Former Female Smokers (%)** |
| Andorra | 32.97% | 22.35% | 30.75% | 15.51% |
| Argentina | 28.07% | 20.78% | 29.46% | 18.68% |
| Australia | 21.15% | 18.09% | 29.64% | 23.19% |
| Austria | 35.54% | 26.78% | 24.69% | 15.87% |
| Bahrain | 19.11% | 4.97% | 19.59% | 2.38% |
| Belgium | 30.97% | 22.59% | 31.14% | 18.97% |
| Brunei Darussalam | 27.20% | 5.60% | 20.43% | 5.87% |
| Canada | 25.19% | 21.38% | 31.51% | 23.93% |
| Chile | 33.97% | 29.31% | 30.20% | 24.04% |
| Croatia | 34.33% | 24.88% | 25.67% | 11.17% |
| Cyprus | 40.44% | 18.64% | 28.64% | 9.79% |
| Czech Republic | 34.19% | 24.01% | 27.48% | 15.52% |
| Denmark | 33.91% | 32.23% | 30.08% | 25.96% |
| Estonia | 37.60% | 17.83% | 29.92% | 12.63% |
| Finland | 25.63% | 18.77% | 32.35% | 17.38% |
| France | 32.21% | 24.33% | 32.66% | 17.13% |
| Germany | 31.31% | 22.28% | 30.30% | 18.02% |
| Greece | 43.07% | 27.07% | 26.80% | 9.53% |
| Hong Kong | 48.57% | 5.15% | 7.55% | 1.14% |
| Hungary | 35.84% | 26.48% | 25.78% | 10.42% |
| Iceland | 22.70% | 20.98% | 31.94% | 15.48% |
| Ireland | 30.19% | 25.28% | 27.38% | 19.23% |
| Israel | 29.29% | 16.52% | 27.65% | 13.94% |
| Italy | 28.91% | 18.36% | 30.91% | 13.70% |
| Japan | 44.92% | 12.55% | 19.29% | 5.66% |
| Kuwait | 31.32% | 4.98% | 20.41% | 1.58% |
| Latvia | 41.41% | 15.95% | 26.61% | 8.47% |
| Lithuania | 37.97% | 11.99% | 25.05% | 7.40% |
| Luxembourg | 29.92% | 23.65% | 31.06% | 17.96% |
| Malta | 27.74% | 17.14% | 25.98% | 10.97% |
| Montenegro | 40.57% | 31.31% | 24.91% | 11.78% |
| Netherlands | 31.94% | 25.74% | 36.16% | 27.86% |
| New Zealand | 23.01% | 23.20% | 37.37% | 29.43% |
| North Korea | 46.83% | 6.53% | 19.03% | 4.67% |
| Norway | 33.71% | 29.90% | 22.37% | 18.71% |
| Poland | 35.08% | 21.47% | 28.37% | 13.72% |
| Portugal | 31.56% | 14.01% | 24.50% | 7.83% |
| Qatar | 19.75% | 2.95% | 14.94% | 1.18% |
| Romania | 38.01% | 18.01% | 23.11% | 6.33% |
| Russia | 50.51% | 12.08% | 24.71% | 6.24% |
| Saudi Arabia | 17.67% | 2.05% | 9.24% | 1.34% |
| Singapore | 21.64% | 7.48% | 12.65% | 3.04% |
| Slovakia | 33.17% | 18.32% | 26.77% | 13.21% |
| Slovenia | 22.77% | 17.21% | 30.53% | 15.17% |
| South Korea | 47.07% | 4.86% | 5.84% | 0.98% |
| Spain | 36.92% | 23.01% | 30.45% | 12.27% |
| Sweden | 16.31% | 20.17% | 32.09% | 28.22% |
| Switzerland | 31.98% | 23.43% | 27.69% | 17.34% |
| Taiwan | 41.57% | 5.46% | 6.14% | 0.96% |
| United Arab Emirates | 20.10% | 3.91% | 18.20% | 2.36% |
| United Kingdom | 25.53% | 24.63% | 45.29% | 34.93% |
| United States of America | 23.19% | 19.72% | 35.64% | 23.03% |
| **High HDI** | | | | |
| Albania | 38.17% | 6.95% | 15.41% | 2.29% |
| Algeria | 29.02% | 1.69% | 28.81% | 1.26% |
| Antigua and Barbuda | 9.75% | 4.40% | 27.88% | 11.30% |
| Armenia | 51.32% | 2.81% | 17.41% | 1.39% |
| Azerbaijan | 41.84% | 1.95% | 14.28% | 0.87% |
| Bahamas | 9.35% | 3.32% | 27.39% | 10.67% |
| Barbados | 16.16% | 4.33% | 24.67% | 5.70% |
| Belarus | 45.22% | 13.63% | 26.47% | 7.09% |
| Belize | 18.88% | 3.82% | 26.22% | 10.65% |
| Bosnia and Herzegovina | 38.36% | 20.65% | 20.71% | 7.79% |
| Brazil | 26.37% | 18.12% | 34.56% | 21.99% |
| Bulgaria | 41.17% | 28.61% | 23.66% | 8.47% |
| China | 48.57% | 5.15% | 7.55% | 1.14% |
| Colombia | 24.49% | 12.20% | 23.99% | 8.83% |
| Cook Islands | 36.15% | 29.44% | 23.42% | 15.51% |
| Costa Rica | 22.09% | 11.05% | 33.26% | 13.09% |
| Cuba | 37.83% | 20.70% | 25.80% | 11.24% |
| Dominica | 12.27% | 5.79% | 33.19% | 8.30% |
| Dominican Republic | 17.62% | 14.74% | 26.34% | 15.35% |
| Ecuador | 21.42% | 5.18% | 26.93% | 10.18% |
| Fiji | 39.57% | 14.20% | 29.53% | 15.45% |
| Georgia | 42.06% | 5.40% | 25.73% | 2.10% |
| Grenada | 16.38% | 5.43% | 26.71% | 11.03% |
| Iran | 20.20% | 3.66% | 17.36% | 3.38% |
| Jamaica | 24.69% | 8.83% | 26.67% | 10.52% |
| Jordan | 39.88% | 9.17% | 20.79% | 4.54% |
| Kazakhstan | 41.23% | 6.44% | 7.45% | 3.07% |
| Lebanon | 39.02% | 21.67% | 16.22% | 7.64% |
| Libya | 30.22% | 1.26% | 12.40% | 0.53% |
| Macao | 48.57% | 5.15% | 7.55% | 1.14% |
| Malaysia | 45.81% | 4.71% | 13.37% | 1.35% |
| Maldives | 41.30% | 7.32% | 16.20% | 2.18% |
| Mauritius | 36.72% | 4.82% | 16.60% | 3.62% |
| Mexico | 33.21% | 12.37% | 28.36% | 11.62% |
| Mongolia | 41.25% | 6.21% | 13.75% | 2.21% |
| Nauru | 40.40% | 38.63% | 22.00% | 17.07% |
| North Macedonia | 41.01% | 25.49% | 18.94% | 8.05% |
| Oman | 12.50% | 2.03% | 13.01% | 2.79% |
| Panama | 17.64% | 6.71% | 14.89% | 7.12% |
| Papua New Guinea | 42.20% | 21.80% | 16.18% | 9.63% |
| Peru | 19.49% | 5.82% | 27.39% | 6.71% |
| Saint Kitts and Nevis | 11.26% | 3.43% | 22.97% | 6.34% |
| Saint Lucia | 20.56% | 5.95% | 26.53% | 10.34% |
| Saint Vincent and the Grenadines | 17.92% | 4.50% | 26.74% | 10.53% |
| Samoa | 41.14% | 14.88% | 10.39% | 4.45% |
| Serbia | 37.47% | 25.50% | 24.85% | 10.82% |
| Seychelles | 40.61% | 7.77% | 19.26% | 3.29% |
| Sri Lanka | 33.64% | 2.52% | 21.14% | 2.43% |
| Suriname | 35.63% | 10.78% | 25.63% | 9.56% |
| Thailand | 43.26% | 5.98% | 25.97% | 3.18% |
| Tonga | 48.48% | 13.41% | 14.27% | 5.71% |
| Trinidad and Tobago | 24.83% | 7.07% | 31.04% | 12.03% |
| Tunisia | 45.46% | 3.13% | 16.05% | 1.92% |
| Turkey | 43.84% | 14.18% | 27.74% | 5.05% |
| Ukraine | 51.40% | 11.42% | 25.32% | 6.61% |
| Uruguay | 34.27% | 24.86% | 33.91% | 19.87% |
| Uzbekistan | 20.75% | 1.59% | 15.58% | 2.25% |
| Venezuela | 22.98% | 12.86% | 26.19% | 9.13% |
| **Medium HDI** | | | | |
| Bangladesh | 41.64% | 3.36% | 28.88% | 4.15% |
| Bhutan | 12.36% | 5.20% | 30.58% | 2.08% |
| Bolivia | 12.43% | 5.83% | 28.17% | 10.90% |
| Botswana | 31.32% | 9.57% | 18.70% | 5.24% |
| Cabo Verde | 11.53% | 3.43% | 13.20% | 4.00% |
| Cambodia | 55.45% | 7.93% | 10.32% | 1.31% |
| Democratic Republic of the Congo | 18.10% | 1.43% | 15.43% | 3.17% |
| Egypt | 35.87% | 1.39% | 16.13% | 0.79% |
| El Salvador | 10.77% | 3.12% | 27.70% | 8.86% |
| Equatorial Guinea | 21.82% | 2.08% | 15.31% | 3.06% |
| Gabon | 16.77% | 2.73% | 15.79% | 3.11% |
| Ghana | 9.20% | 1.93% | 14.28% | 1.43% |
| Guatemala | 19.96% | 4.49% | 26.42% | 8.27% |
| Guyana | 19.92% | 4.33% | 26.50% | 10.49% |
| Honduras | 25.27% | 5.08% | 26.09% | 6.75% |
| India | 31.86% | 4.65% | 8.74% | 1.59% |
| Indonesia | 55.66% | 4.16% | 12.36% | 1.58% |
| Iraq | 36.77% | 4.77% | 23.14% | 5.19% |
| Kenya | 26.17% | 5.50% | 12.30% | 2.95% |
| Kiribati | 58.23% | 36.20% | 21.32% | 13.66% |
| Kyrgyzstan | 35.32% | 2.89% | 14.24% | 1.25% |
| Laos | 42.29% | 5.55% | 15.72% | 2.35% |
| Micronesia (Federated States of) | 53.05% | 28.33% | 20.63% | 12.03% |
| Moldova | 34.62% | 5.46% | 31.62% | 3.29% |
| Morocco | 24.55% | 1.24% | 18.87% | 0.73% |
| Myanmar | 47.39% | 18.32% | 17.33% | 5.74% |
| Namibia | 28.84% | 17.51% | 11.56% | 5.47% |
| Nepal | 48.91% | 32.93% | 14.06% | 10.84% |
| Nicaragua | 21.42% | 5.22% | 26.45% | 7.13% |
| Niue | 25.55% | 13.35% | 22.92% | 8.54% |
| Pakistan | 37.68% | 6.46% | 5.63% | 1.44% |
| Paraguay | 37.51% | 14.05% | 31.35% | 21.63% |
| Philippines | 48.94% | 15.36% | 24.77% | 8.50% |
| Sao Tome and Principe | 8.37% | 2.27% | 19.76% | 3.57% |
| South Africa | 34.63% | 13.02% | 8.97% | 4.48% |
| Sudan | 20.99% | 2.56% | 16.79% | 1.30% |
| Swaziland | 34.63% | 13.02% | 8.97% | 4.48% |
| Tajikistan | 21.41% | 1.46% | 15.88% | 1.98% |
| Timor-Leste | 58.27% | 5.87% | 8.49% | 2.19% |
| Turkmenistan | 29.80% | 3.85% | 15.82% | 1.80% |
| Tuvalu | 39.47% | 17.00% | 21.68% | 9.04% |
| Vanuatu | 39.95% | 7.24% | 26.85% | 3.49% |
| Vietnam | 52.69% | 4.87% | 24.77% | 1.93% |
| Zambia | 23.51% | 9.65% | 12.19% | 1.98% |
| **Low HDI** | | | | |
| Afghanistan | 9.35% | 1.63% | 18.08% | 3.05% |
| Angola | 16.33% | 2.73% | 15.67% | 3.10% |
| Benin | 16.48% | 3.20% | 5.75% | 1.63% |
| Burkina Faso | 17.19% | 2.19% | 17.45% | 1.48% |
| Burundi | 15.46% | 4.91% | 19.23% | 2.76% |
| Cameroon | 17.78% | 2.05% | 11.61% | 0.85% |
| Central African Republic | 14.77% | 1.82% | 15.79% | 3.15% |
| Chad | 19.88% | 4.54% | 12.62% | 1.61% |
| Comoros | 27.48% | 3.40% | 19.38% | 2.81% |
| Côte d'Ivoire | 20.10% | 3.45% | 13.10% | 1.36% |
| Djibouti | 34.95% | 5.01% | 11.89% | 3.58% |
| Eritrea | 12.47% | 0.80% | 13.99% | 0.72% |
| Eswatini | 17.29% | 5.16% | 12.26% | 1.92% |
| Ethiopia | 8.86% | 1.14% | 8.20% | 1.12% |
| Guinea | 19.09% | 2.07% | 11.80% | 1.36% |
| Guinea-Bissau | 5.12% | 0.85% | 13.57% | 1.54% |
| Haiti | 13.32% | 4.15% | 26.73% | 11.42% |
| Lesotho | 26.74% | 2.96% | 11.97% | 2.68% |
| Liberia | 14.02% | 2.48% | 16.50% | 2.71% |
| Madagascar | 26.61% | 3.36% | 12.81% | 2.37% |
| Malawi | 25.06% | 4.81% | 16.06% | 3.15% |
| Mali | 18.46% | 2.18% | 12.42% | 1.54% |
| Mauritania | 27.53% | 8.28% | 12.70% | 1.51% |
| Mozambique | 21.86% | 6.65% | 12.79% | 2.84% |
| Niger | 14.72% | 1.78% | 13.17% | 1.36% |
| Nigeria | 9.14% | 1.97% | 8.25% | 1.11% |
| Republic of the Congo | 13.72% | 1.68% | 15.87% | 3.09% |
| Rwanda | 24.08% | 11.55% | 12.50% | 3.53% |
| Senegal | 22.27% | 2.42% | 17.53% | 0.61% |
| Sierra Leone | 29.51% | 8.76% | 12.48% | 4.52% |
| Solomon Islands | 40.27% | 14.77% | 21.43% | 8.26% |
| Somalia | 21.02% | 3.64% | 12.89% | 2.49% |
| Syria | 41.73% | 6.62% | 16.18% | 2.38% |
| Tanzania | 27.37% | 4.89% | 48.59% | 36.59% |
| The Gambia | 24.94% | 1.25% | 18.31% | 1.51% |
| Togo | 17.85% | 3.00% | 13.76% | 1.53% |
| Uganda | 22.04% | 6.68% | 10.47% | 7.13% |
| Yemen | 28.14% | 7.96% | 17.23% | 2.70% |
| Zimbabwe | 29.55% | 5.78% | 17.30% | 2.39% |

**Data is unavailable for the following countries:*

*-Very High HDI (New Caledonia)*

*-High HDI (French Guiana, Guadeloupe (High HDI)*

*-Low HDI (French Polynesia, Reunion).**

| **Table S2. Countries categorized by World Bank HDI and the PAF for incidence and mortality in each country for current and former smokers.** | | | | |
| --- | --- | --- | --- | --- |
| **Very High** | | | | |
| **Countries** | **Incidence PAF (Current Smokers)** | **Incidence PAF (Former Smokers)** | **Mortality PAF (Current Smokers)** | **Mortality PAF (Former Smokers)** |
| Argentina | 10.6% | 4.6% | 14.1% | 5.9% |
| Australia | 7.8% | 4.5% | 12.8% | 7.2% |
| Austria | 13.7% | 3.7% | 19.2% | 5.1% |
| Bahrain | 6.0% | 2.4% | 8.8% | 3.5% |
| Belgium | 12.3% | 4.9% | 18.0% | 6.9% |
| Brunei Darussalam | 8.5% | 3.1% | 12.5% | 4.4% |
| Canada | 11.9% | 5.9% | 16.6% | 8.0% |
| Chile | 10.8% | 4.2% | 14.9% | 5.7% |
| Croatia | 14.1% | 3.9% | 20.1% | 5.4% |
| Cyprus | 11.7% | 3.3% | 17.3% | 4.7% |
| Czech Republic | 12.5% | 3.9% | 17.9% | 5.5% |
| Denmark | 13.2% | 4.9% | 19.5% | 6.8% |
| Estonia | 12.2% | 4.0% | 16.4% | 5.3% |
| Finland | 8.5% | 4.1% | 13.7% | 6.4% |
| France | 12.6% | 4.8% | 17.7% | 6.7% |
| Germany | 12.6% | 4.8% | 16.9% | 6.2% |
| Greece | 16.8% | 4.0% | 21.8% | 5.0% |
| Hungary | 17.6% | 4.3% | 24.3% | 5.7% |
| Iceland | 10.4% | 4.8% | 13.7% | 6.2% |
| Ireland | 11.7% | 4.3% | 17.8% | 6.4% |
| Israel | 9.9% | 3.8% | 13.2% | 4.9% |
| Italy | 11.9% | 5.0% | 15.7% | 6.2% |
| Japan | 16.4% | 3.1% | 20.1% | 3.7% |
| Kuwait | 6.7% | 1.9% | 9.9% | 2.7% |
| Latvia | 12.6% | 3.2% | 17.4% | 4.4% |
| Lithuania | 11.7% | 3.1% | 16.8% | 4.5% |
| Luxembourg | 11.3% | 4.5% | 19.3% | 7.3% |
| Malta | 9.0% | 3.3% | 14.9% | 5.3% |
| Montenegro | 18.0% | 4.1% | 22.5% | 5.0% |
| Netherlands | 13.0% | 6.3% | 18.1% | 8.4% |
| New Zealand | 8.5% | 5.5% | 13.4% | 8.2% |
| North Korea | 19.5% | 4.1% | 22.7% | 4.7% |
| Norway | 11.4% | 3.2% | 17.6% | 4.8% |
| Poland | 16.0% | 4.9% | 19.9% | 6.0% |
| Portugal | 11.6% | 3.6% | 15.4% | 4.7% |
| Qatar | 5.9% | 1.9% | 8.6% | 2.8% |
| Romania | 15.8% | 3.6% | 20.5% | 4.6% |
| Russia | 14.8% | 3.1% | 19.2% | 4.0% |
| Saudi Arabia | 4.9% | 1.2% | 7.1% | 1.7% |
| Singapore | 9.7% | 2.3% | 14.7% | 3.4% |
| Slovakia | 12.4% | 4.0% | 16.2% | 5.2% |
| Slovenia | 10.7% | 5.2% | 14.1% | 6.7% |
| South Korea | 15.1% | 0.9% | 22.5% | 1.3% |
| Spain | 14.3% | 4.6% | 19.7% | 6.2% |
| Sweden | 6.7% | 4.8% | 10.5% | 7.1% |
| Switzerland | 12.2% | 4.2% | 17.2% | 5.7% |
| United Arab Emirates | 5.0% | 1.9% | 7.9% | 2.9% |
| United Kingdom | 11.0% | 7.5% | 15.0% | 10.2% |
| United States of America | 10.3% | 5.8% | 14.9% | 8.3% |
| **High HDI** | | | | |
| Albania | 18.7% | 3.0% | 22.9% | 3.6% |
| Algeria | 7.8% | 3.3% | 10.9% | 4.4% |
| Armenia | 15.7% | 2.3% | 19.4% | 2.8% |
| Azerbaijan | 14.9% | 2.3% | 18.2% | 2.7% |
| Bahamas | 2.4% | 3.2% | 3.2% | 4.2% |
| Barbados | 4.1% | 2.5% | 4.7% | 2.9% |
| Belarus | 14.6% | 3.5% | 20.2% | 4.7% |
| Belize | 5.1% | 3.9% | 7.8% | 5.7% |
| Bosnia and Herzegovina | 18.5% | 3.9% | 22.0% | 4.6% |
| Brazil | 9.0% | 4.9% | 13.5% | 7.3% |
| Bulgaria | 16.5% | 3.5% | 20.6% | 4.3% |
| China | 20.1% | 1.5% | 27.1% | 1.9% |
| Colombia | 6.9% | 2.7% | 10.0% | 3.8% |
| Costa Rica | 5.6% | 3.6% | 7.4% | 4.8% |
| Cuba | 18.2% | 4.8% | 21.4% | 5.7% |
| Dominican Republic | 7.4% | 4.1% | 9.3% | 5.2% |
| Ecuador | 4.5% | 2.9% | 6.0% | 3.9% |
| Fiji | 7.1% | 2.9% | 9.3% | 3.8% |
| Georgia | 13.7% | 3.4% | 16.6% | 4.1% |
| Iran | 7.8% | 3.1% | 9.0% | 3.5% |
| Jamaica | 6.2% | 3.0% | 7.7% | 3.7% |
| Jordan | 11.0% | 2.5% | 15.3% | 3.4% |
| Kazakhstan | 15.5% | 1.4% | 19.4% | 1.7% |
| Lebanon | 13.4% | 2.2% | 18.4% | 3.0% |
| Libya | 11.6% | 2.0% | 14.4% | 2.5% |
| Malaysia | 12.8% | 1.6% | 16.2% | 2.0% |
| Maldives | 12.7% | 2.1% | 17.3% | 2.9% |
| Mauritius | 8.6% | 1.9% | 12.6% | 2.6% |
| Mexico | 6.4% | 2.6% | 9.6% | 3.9% |
| Mongolia | 14.8% | 2.8% | 16.3% | 3.1% |
| North Macedonia | 17.3% | 3.1% | 22.7% | 4.0% |
| Oman | 4.1% | 1.9% | 5.2% | 2.5% |
| Panama | 4.5% | 1.8% | 6.9% | 2.7% |
| Papua New Guinea | 14.0% | 2.2% | 15.0% | 2.6% |
| Peru | 4.2% | 2.4% | 5.8% | 3.3% |
| Saint Lucia | 5.3% | 2.9% | 6.8% | 3.8% |
| Samoa | 14.0% | 1.6% | 18.6% | 2.0% |
| Serbia | 18.0% | 4.4% | 22.7% | 5.4% |
| Sri Lanka | 11.5% | 2.8% | 13.4% | 3.5% |
| Suriname | 9.3% | 3.1% | 12.3% | 3.9% |
| Thailand | 14.0% | 3.9% | 16.9% | 4.8% |
| Trinidad and Tobago | 6.1% | 3.4% | 7.8% | 4.4% |
| Tunisia | 17.2% | 2.6% | 21.6% | 3.2% |
| Turkey | 17.8% | 4.3% | 25.1% | 5.9% |
| Ukraine | 15.9% | 3.4% | 20.5% | 4.2% |
| Uruguay | 13.0% | 5.2% | 16.8% | 6.5% |
| Uzbekistan | 6.7% | 2.2% | 8.0% | 2.7% |
| Venezuela | 8.8% | 3.6% | 11.8% | 4.8% |
| **Medium HDI** | | | | |
| Bangladesh | 19.2% | 5.5% | 20.1% | 6.3% |
| Bhutan | 7.8% | 5.9% | 8.5% | 6.7% |
| Bolivia | 4.4% | 3.9% | 5.1% | 4.5% |
| Botswana | 7.9% | 1.9% | 9.6% | 2.5% |
| Cabo Verde | 3.8% | 1.9% | 4.4% | 2.4% |
| Cambodia | 15.7% | 1.4% | 18.4% | 1.7% |
| Democratic Republic of the Congo | 2.8% | 1.4% | 3.3% | 1.7% |
| Egypt | 9.1% | 2.1% | 11.1% | 2.7% |
| El Salvador | 2.4% | 2.8% | 3.4% | 4.0% |
| Equatorial Guinea | 4.0% | 1.5% | 4.7% | 1.9% |
| Gabon | 4.2% | 1.8% | 4.7% | 2.2% |
| Ghana | 2.2% | 1.6% | 2.6% | 1.9% |
| Guatemala | 3.8% | 2.9% | 5.3% | 4.0% |
| Guyana | 3.2% | 2.3% | 4.5% | 3.1% |
| Honduras | 5.8% | 3.0% | 6.0% | 3.1% |
| India | 13.7% | 1.4% | 14.7% | 1.6% |
| Indonesia | 12.5% | 1.3% | 16.4% | 1.7% |
| Iraq | 8.3% | 2.4% | 11.4% | 3.3% |
| Kenya | 5.5% | 1.3% | 6.4% | 1.6% |
| Kyrgyzstan | 12.1% | 2.1% | 14.3% | 2.5% |
| Laos | 13.9% | 2.5% | 16.8% | 3.1% |
| Moldova | 13.1% | 4.9% | 16.3% | 6.0% |
| Morocco | 10.4% | 3.2% | 14.8% | 4.4% |
| Myanmar | 19.7% | 3.0% | 22.4% | 3.5% |
| Namibia | 7.3% | 1.1% | 8.5% | 1.3% |
| Nepal | 18.8% | 2.3% | 21.4% | 2.6% |
| Nicaragua | 4.4% | 2.7% | 6.2% | 3.7% |
| Pakistan | 12.6% | 0.8% | 14.4% | 0.9% |
| Paraguay | 9.2% | 4.0% | 13.0% | 5.4% |
| Philippines | 14.0% | 3.2% | 18.5% | 4.2% |
| Sao Tome and Principe | 4.6% | 4.5% | 6.7% | 6.5% |
| South Africa | 11.4% | 1.4% | 13.9% | 1.7% |
| Sudan | 3.9% | 1.4% | 4.6% | 1.7% |
| Tajikistan | 5.1% | 1.8% | 6.5% | 2.3% |
| Timor-Leste | 14.2% | 1.1% | 18.5% | 1.4% |
| Turkmenistan | 10.0% | 2.3% | 12.0% | 2.8% |
| Vanuatu | 8.0% | 2.5% | 12.5% | 3.9% |
| Vietnam | 16.8% | 3.7% | 20.6% | 4.6% |
| Zambia | 5.0% | 0.8% | 5.6% | 1.0% |
| **Low** | | | | |
| Afghanistan | 3.9% | 3.0% | 4.6% | 3.6% |
| Angola | 3.5% | 1.6% | 3.9% | 1.9% |
| Benin | 3.3% | 0.7% | 3.8% | 0.8% |
| Burkina Faso | 3.6% | 1.7% | 3.8% | 1.9% |
| Burundi | 4.0% | 1.9% | 4.6% | 2.2% |
| Cameroon | 3.2% | 0.8% | 3.9% | 1.0% |
| Central African Republic | 2.4% | 1.3% | 2.6% | 1.5% |
| Chad | 4.0% | 1.2% | 4.6% | 1.4% |
| Comoros | 5.3% | 1.9% | 6.2% | 2.4% |
| Côte d'Ivoire | 2.8% | 0.8% | 3.3% | 1.0% |
| Djibouti | 5.9% | 1.2% | 6.6% | 1.3% |
| Eritrea | 2.2% | 1.2% | 2.5% | 1.3% |
| Eswatini | 3.7% | 0.8% | 4.7% | 1.1% |
| Ethiopia | 1.9% | 0.8% | 2.3% | 1.0% |
| Guinea | 3.8% | 1.2% | 4.5% | 1.5% |
| Guinea-Bissau | 1.1% | 1.3% | 1.3% | 1.7% |
| Haiti | 3.3% | 3.3% | 3.8% | 3.9% |
| Lesotho | 4.6% | 1.2% | 5.4% | 1.4% |
| Liberia | 2.7% | 1.6% | 3.3% | 2.0% |
| Madagascar | 5.3% | 1.2% | 6.0% | 1.4% |
| Malawi | 4.4% | 1.5% | 5.0% | 1.7% |
| Mali | 3.9% | 1.2% | 4.3% | 1.4% |
| Mauritania | 5.5% | 1.0% | 6.4% | 1.2% |
| Mozambique | 4.2% | 1.1% | 4.6% | 1.2% |
| Niger | 2.8% | 1.3% | 3.1% | 1.5% |
| Nigeria | 1.5% | 0.5% | 1.7% | 0.7% |
| Republic of the Congo | 2.0% | 1.4% | 2.5% | 1.8% |
| Rwanda | 6.8% | 1.4% | 7.7% | 1.6% |
| Senegal | 3.8% | 1.3% | 4.4% | 1.6% |
| Sierra Leone | 6.7% | 1.6% | 8.1% | 2.0% |
| Solomon Islands | 7.8% | 2.0% | 11.8% | 3.1% |
| Somalia | 4.1% | 1.2% | 4.6% | 1.4% |
| Syria | 12.1% | 1.9% | 16.1% | 2.5% |
| Tanzania | 5.1% | 7.6% | 5.9% | 8.7% |
| The Gambia | 6.3% | 2.7% | 7.8% | 3.3% |
| Togo | 3.4% | 1.2% | 3.9% | 1.4% |
| Uganda | 5.1% | 1.7% | 5.9% | 2.0% |
| Yemen | 7.7% | 2.0% | 9.1% | 2.4% |
| Zimbabwe | 4.9% | 1.3% | 5.9% | 1.6% |

**Data is unavailable for the following countries:*

*-Very High HDI (Andorra, Hong Kong, New Caledonia, Taiwan)*

*-High HDI (Antigua and Barbuda, Cook Islands, Dominica, French Guiana, Grenada, Guadeloupe, Macao, Nauru, Saint Kitts and Nevis, Saint Vincent and the Grenadines, Seychelles, Tonga)*

*-Medium HDI (Kiribati, Micronesia (Federated States of), Niue, Swaziland, Tuvalu)*

*-Low HDI (French Polynesia, Reunion).**

| **Table S3. Number of cancer incidence cases attributable to tobacco smoking in current smokers characterized by global, very high, high, medium, and low HDI categories (rounded to nearest whole number).** | | | | | | |
| --- | --- | --- | --- | --- | --- | --- |
|  |  | **Attributable Cases** | | | | |
| **Cancers** | **Sex** | **Global** | **HDI Category** | | | |
|  |  |  | **Very High** | **High** | **Medium** | **Low** |
| Bladder | Male | 160672 | 86560 | 53077 | 19266 | 1769 |
|  | Female | 26225 | 20223 | 4554 | 1144 | 304 |
| Breast | Female | 30416 | 20691 | 6394 | 2982 | 350 |
| Cervix | Female | 37733 | 10831 | 13055 | 11305 | 2541 |
| Colorectal | Male | 52964 | 21525 | 24841 | 6196 | 402 |
|  | Female | 14687 | 10346 | 3480 | 780 | 81 |
| Esophagus | Male | 124995 | 21760 | 74545 | 26491 | 2199 |
|  | Female | 13379 | 4590 | 5055 | 3233 | 501 |
| Kidney | Male | 40569 | 22167 | 14178 | 3802 | 422 |
|  | Female | 10158 | 7800 | 1839 | 441 | 77 |
| Larynx | Male | 92441 | 24806 | 33442 | 32676 | 1516 |
|  | Female | 7139 | 3291 | 1969 | 1756 | 123 |
| Liver | Male | 106436 | 20479 | 63858 | 20414 | 1685 |
|  | Female | 11647 | 5303 | 4580 | 1515 | 248 |
| Lung | Male | 1061270 | 335270 | 599980 | 121128 | 4892 |
|  | Female | 344503 | 179020 | 143244 | 21056 | 1184 |
| Mucinous Ovary | Female | 2371 | 1345 | 592 | 399 | 34 |
| Myeloid Leukemia | Male | 3534 | 1292 | 1280 | 895 | 66 |
|  | Female | 984 | 652 | 206 | 114 | 12 |
| Nasopharynx | Male | 29066 | 1819 | 15763 | 10955 | 530 |
|  | Female | 2407 | 409 | 1099 | 823 | 75 |
| Oral Cavity | Male | 116766 | 28133 | 23885 | 63366 | 1383 |
|  | Female | 20982 | 10831 | 3903 | 5979 | 269 |
| Pancreatic | Male | 53630 | 25005 | 23231 | 4879 | 514 |
|  | Female | 20220 | 15128 | 4221 | 779 | 92 |
| Pharynx | Male | 96601 | 33252 | 20792 | 41555 | 1002 |
|  | Female | 11093 | 6150 | 1607 | 3214 | 121 |
| Sinonasal | Male | 1714 | 530 | 683 | 478 | 23 |
|  | Female | 316 | 171 | 80 | 61 | 3 |
| Stomach | Male | 125615 | 39492 | 69446 | 15586 | 1089 |
|  | Female | 18828 | 10207 | 6571 | 1841 | 208 |

| **Table S4. Number of cancer mortality cases attributable to tobacco smoking in current smokers characterized by global, very high, high, medium, and low HDI categories (rounded to nearest whole number).** | | | | | | |
| --- | --- | --- | --- | --- | --- | --- |
|  |  | **Attributable Cases** | | | | |
| **Cancers** | **Sex** | **Global** | **HDI Category** | | | |
|  |  |  | **Very High** | **High** | **Medium** | **Low** |
| Bladder | Male | 58406 | 24739 | 22405 | 10251 | 1012 |
|  | Female | 9231 | 6590 | 1860 | 608 | 172 |
| Breast | Female | 7657 | 4490 | 1693 | 1290 | 184 |
| Cervix | Female | 19195 | 4824 | 5921 | 6764 | 1687 |
| Colorectal | Male | 24773 | 9225 | 11837 | 3426 | 284 |
|  | Female | 6636 | 4422 | 1709 | 447 | 58 |
| Esophagus | Male | 108070 | 17687 | 63150 | 25160 | 2072 |
|  | Female | 11465 | 3685 | 4283 | 3025 | 472 |
| Kidney | Male | 14823 | 7044 | 5355 | 2155 | 269 |
|  | Female | 3395 | 2451 | 649 | 246 | 49 |
| Larynx | Male | 50882 | 10336 | 18946 | 20542 | 1058 |
|  | Female | 3627 | 1259 | 1158 | 1132 | 78 |
| Liver | Male | 92584 | 15907 | 55490 | 19591 | 1597 |
|  | Female | 10265 | 4487 | 4107 | 1436 | 235 |
| Lung | Male | 835310 | 240926 | 480225 | 109566 | 4593 |
|  | Female | 225675 | 119087 | 86414 | 19070 | 1105 |
| Mucinous Ovary | Female | 1532 | 872 | 365 | 272 | 23 |
| Myeloid Leukemia | Male | 2254 | 704 | 843 | 657 | 49 |
|  | Female | 580 | 345 | 138 | 87 | 9 |
| Nasopharynx | Male | 18237 | 960 | 9309 | 7598 | 371 |
|  | Female | 1336 | 201 | 565 | 517 | 53 |
| Oral Cavity | Male | 57991 | 9053 | 12461 | 35548 | 929 |
|  | Female | 8638 | 3274 | 1780 | 3401 | 183 |
| Pancreatic | Male | 49361 | 22783 | 21394 | 4706 | 478 |
|  | Female | 18491 | 13798 | 3873 | 733 | 86 |
| Pharynx | Male | 48024 | 14254 | 12335 | 20765 | 671 |
|  | Female | 4937 | 2456 | 869 | 1533 | 79 |
| Sinonasal | Male | 1034 | 277 | 399 | 343 | 16 |
|  | Female | 169 | 84 | 41 | 41 | 2 |
| Stomach | Male | 84772 | 18502 | 51765 | 13535 | 971 |
|  | Female | 12299 | 5736 | 4796 | 1582 | 184 |

| **Table S5. Number of cancer incidence cases attributable to tobacco smoking in former smokers characterized by global, very high, high, medium, and low HDI categories (rounded to nearest whole number).** | | | | | | |
| --- | --- | --- | --- | --- | --- | --- |
|  |  | **Attributable Cases** | | | | |
| **Cancers** | **Sex** | **Global** | **HDI Category** | | | |
|  |  |  | **Very High** | **High** | **Medium** | **Low** |
| Bladder | Male | 46309 | 34533 | 8362 | 2818 | 597 |
|  | Female | 8589 | 7101 | 1104 | 207 | 177 |
| Breast | Female | 20067 | 15134 | 3509 | 1106 | 317 |
| Cervix | Female | 7919 | 2630 | 2334 | 1581 | 1374 |
| Colorectal | Male | 33784 | 21868 | 8832 | 2676 | 408 |
|  | Female | 13405 | 10446 | 2382 | 446 | 131 |
| Esophagus | Male | 32453 | 12773 | 11181 | 7049 | 1449 |
|  | Female | 5564 | 2814 | 1232 | 1036 | 483 |
| Kidney | Male | 13519 | 10151 | 2505 | 685 | 178 |
|  | Female | 3976 | 3307 | 515 | 103 | 52 |
| Larynx | Male | 29587 | 13134 | 8771 | 6943 | 740 |
|  | Female | 3116 | 1718 | 849 | 472 | 77 |
| Liver | Male | 34588 | 13928 | 12621 | 6876 | 1163 |
|  | Female | 6438 | 3777 | 1811 | 636 | 213 |
| Lung | Male | 174817 | 104009 | 53265 | 16179 | 1364 |
|  | Female | 78113 | 57161 | 17160 | 3324 | 468 |
| Mucinous Ovary | Female | 151 | 102 | 30 | 15 | 4 |
| Myeloid Leukemia | Male | 6417 | 3906 | 1421 | 933 | 157 |
|  | Female | 2328 | 1763 | 371 | 159 | 36 |
| Nasopharynx | Male | 2864 | 483 | 1118 | 1126 | 137 |
|  | Female | 363 | 107 | 111 | 112 | 33 |
| Oral Cavity | Male | 9801 | 4548 | 1788 | 3249 | 217 |
|  | Female | 2386 | 1549 | 380 | 403 | 54 |
| Pancreatic | Male | 8172 | 5623 | 1953 | 485 | 111 |
|  | Female | 3955 | 3219 | 607 | 102 | 27 |
| Pharynx | Male | 12723 | 6925 | 2231 | 3366 | 201 |
|  | Female | 1774 | 1171 | 250 | 313 | 40 |
| Sinonasal | Male | 678 | 370 | 179 | 119 | 10 |
|  | Female | 178 | 120 | 36 | 20 | 2 |
| Stomach | Male | 23594 | 11156 | 9026 | 2936 | 476 |
|  | Female | 5422 | 3318 | 1576 | 420 | 109 |

| **Table S6. Number of cancer mortality cases attributable to tobacco smoking in former smokers characterized by global, very high, high, medium, and low HDI categories (rounded to nearest whole number).** | | | | | | |
| --- | --- | --- | --- | --- | --- | --- |
|  |  | **Attributable Cases** | | | | |
| **Cancers** | **Sex** | **Global** | **HDI Category** | | | |
|  |  |  | **Very High** | **High** | **Medium** | **Low** |
| Bladder | Male | 14523 | 9372 | 3313 | 1500 | 337 |
|  | Female | 2888 | 2248 | 434 | 109 | 98 |
| Breast | Female | 4686 | 3129 | 933 | 459 | 165 |
| Cervix | Female | 4146 | 1161 | 1165 | 939 | 881 |
| Colorectal | Male | 15371 | 9251 | 4356 | 1478 | 287 |
|  | Female | 5858 | 4336 | 1177 | 254 | 91 |
| Esophagus | Male | 28747 | 10904 | 9758 | 6718 | 1368 |
|  | Female | 4826 | 2314 | 1078 | 977 | 458 |
| Kidney | Male | 4598 | 3083 | 1014 | 386 | 114 |
|  | Female | 1280 | 991 | 200 | 57 | 33 |
| Larynx | Male | 15347 | 5301 | 5188 | 4340 | 518 |
|  | Female | 1445 | 643 | 441 | 313 | 48 |
| Liver | Male | 29726 | 10779 | 11275 | 6568 | 1104 |
|  | Female | 5662 | 3199 | 1661 | 601 | 202 |
| Lung | Male | 134198 | 73897 | 44364 | 14655 | 1282 |
|  | Female | 52269 | 36987 | 11855 | 3006 | 421 |
| Mucinous Ovary | Female | 99 | 66 | 19 | 10 | 3 |
| Myeloid Leukemia | Male | 3749 | 1955 | 994 | 683 | 117 |
|  | Female | 1286 | 877 | 261 | 121 | 27 |
| Nasopharynx | Male | 1785 | 250 | 670 | 770 | 96 |
|  | Female | 204 | 51 | 59 | 70 | 24 |
| Oral Cavity | Male | 4047 | 1245 | 836 | 1819 | 147 |
|  | Female | 854 | 423 | 166 | 229 | 37 |
| Pancreatic | Male | 7477 | 5081 | 1827 | 466 | 103 |
|  | Female | 3596 | 2904 | 569 | 96 | 26 |
| Pharynx | Male | 5749 | 2652 | 1275 | 1691 | 132 |
|  | Female | 763 | 451 | 133 | 151 | 27 |
| Sinonasal | Male | 387 | 192 | 103 | 85 | 7 |
|  | Female | 92 | 58 | 19 | 13 | 2 |
| Stomach | Male | 15729 | 5889 | 6887 | 2531 | 422 |
|  | Female | 3545 | 1906 | 1186 | 359 | 94 |

*Of note, cancer incidence and mortality data for certain countries, such as Afghanistan, were sourced indirectly due to a lack of specific national data. In these cases, data from neighboring countries, such as Pakistan or other neighboring countries, were adapted to approximate the metrics for Afghanistan. This adaptation involved scaling and adjusting the data based on known demographic and population differences to more accurately reflect Afghanistan’s context (1, 2).

| **Table S7. Total cancer cases and PAF including all cancer types related to tobacco smoking for incidence and mortality by country.** | | | | |
| --- | --- | --- | --- | --- |
| **Country** | **Total Cancer Incidence Cases** | **Total Cancer Mortality Cases** | **Incidence PAF** | **Mortality PAF** |
| Afghanistan | 1631 | 1383 | 6.9% | 8.3% |
| Albania | 1673 | 1297 | 21.7% | 26.5% |
| Algeria | 7021 | 5396 | 11.1% | 15.3% |
| Angola | 1225 | 900 | 5.1% | 5.8% |
| Argentina | 19726 | 13992 | 15.2% | 20.0% |
| Armenia | 1695 | 1296 | 18.0% | 22.3% |
| Australia | 18591 | 10192 | 12.3% | 19.9% |
| Austria | 7948 | 5398 | 17.5% | 24.3% |
| Azerbaijan | 3116 | 2349 | 17.1% | 20.9% |
| Bahamas | 52 | 41 | 5.6% | 7.4% |
| Bahrain | 116 | 75 | 8.4% | 12.3% |
| Bangladesh | 41057 | 30575 | 24.7% | 26.3% |
| Barbados | 74 | 56 | 6.7% | 7.6% |
| Belarus | 8159 | 5628 | 18.1% | 25.0% |
| Belgium | 12454 | 7203 | 17.1% | 24.9% |
| Belize | 34 | 29 | 9.0% | 13.5% |
| Benin | 292 | 232 | 4.0% | 4.6% |
| Bhutan | 87 | 73 | 13.8% | 15.2% |
| Bolivia | 1404 | 1045 | 8.3% | 9.6% |
| Bosnia and Herzegovina | 3083 | 2266 | 22.4% | 26.6% |
| Botswana | 218 | 153 | 9.8% | 12.1% |
| Brazil | 82222 | 57500 | 13.9% | 20.9% |
| Brunei Darussalam | 105 | 78 | 11.7% | 16.9% |
| Bulgaria | 6279 | 4636 | 19.9% | 24.9% |
| Burkina Faso | 748 | 615 | 5.2% | 5.6% |
| Burundi | 469 | 391 | 6.0% | 6.8% |
| Cabo Verde | 24 | 19 | 5.7% | 6.7% |
| Cambodia | 3331 | 2754 | 17.1% | 20.1% |
| Cameroon | 783 | 627 | 4.1% | 4.9% |
| Canada | 41167 | 24534 | 17.7% | 24.6% |
| Central African Republic | 98 | 82 | 3.7% | 4.1% |
| Chad | 523 | 436 | 5.2% | 6.0% |
| Chile | 8575 | 6410 | 15.0% | 20.6% |
| China | 1029811 | 742598 | 21.6% | 29.0% |
| Colombia | 11002 | 7772 | 9.6% | 13.9% |
| Comoros | 43 | 36 | 7.2% | 8.6% |
| Costa Rica | 1084 | 732 | 9.2% | 12.2% |
| Côte d'Ivoire | 765 | 603 | 3.6% | 4.3% |
| Croatia | 4963 | 3498 | 18.0% | 25.5% |
| Cuba | 10517 | 7402 | 23.0% | 27.1% |
| Cyprus | 889 | 617 | 15.0% | 22.0% |
| Czech Republic | 10077 | 6522 | 16.5% | 23.4% |
| Democratic Republic of the Congo | 2221 | 1861 | 4.3% | 5.0% |
| Denmark | 7932 | 4493 | 18.0% | 26.3% |
| Djibouti | 56 | 44 | 7.1% | 7.9% |
| Dominican Republic | 2287 | 1691 | 11.5% | 14.5% |
| Ecuador | 2206 | 1568 | 7.5% | 9.9% |
| Egypt | 16748 | 12984 | 11.3% | 13.8% |
| El Salvador | 497 | 389 | 5.2% | 7.4% |
| Equatorial Guinea | 50 | 38 | 5.5% | 6.6% |
| Eritrea | 82 | 65 | 3.4% | 3.8% |
| Estonia | 1264 | 878 | 16.2% | 21.7% |
| Eswatini | 49 | 40 | 4.5% | 5.8% |
| Ethiopia | 2127 | 1750 | 2.7% | 3.3% |
| Fiji | 158 | 113 | 9.9% | 13.1% |
| Finland | 4439 | 2684 | 12.6% | 20.0% |
| France | 75889 | 46091 | 17.4% | 24.3% |
| Gabon | 111 | 76 | 6.0% | 7.0% |
| Georgia | 2253 | 1705 | 17.1% | 20.7% |
| Germany | 92106 | 58428 | 17.4% | 23.2% |
| Ghana | 1008 | 804 | 3.7% | 4.5% |
| Greece | 13107 | 8615 | 20.7% | 26.7% |
| Guatemala | 1157 | 952 | 6.7% | 9.3% |
| Guinea | 429 | 379 | 5.0% | 6.0% |
| Guinea-Bissau | 28 | 25 | 2.4% | 3.0% |
| Guyana | 67 | 46 | 5.5% | 7.6% |
| Haiti | 909 | 692 | 6.6% | 7.7% |
| Honduras | 931 | 637 | 8.7% | 9.1% |
| Hungary | 13666 | 9675 | 21.9% | 30.0% |
| Iceland | 253 | 133 | 15.2% | 19.9% |
| India | 211393 | 148903 | 15.1% | 16.3% |
| Indonesia | 55315 | 43408 | 13.8% | 18.0% |
| Iran | 14522 | 10871 | 10.9% | 12.5% |
| Iraq | 3957 | 3150 | 10.7% | 14.7% |
| Ireland | 4307 | 2492 | 16.0% | 24.2% |
| Israel | 4160 | 2295 | 13.8% | 18.1% |
| Italy | 68897 | 42144 | 16.9% | 21.9% |
| Jamaica | 681 | 527 | 9.2% | 11.4% |
| Japan | 193474 | 101078 | 19.5% | 23.8% |
| Jordan | 1638 | 1199 | 13.5% | 18.7% |
| Kazakhstan | 5763 | 4339 | 16.9% | 21.1% |
| Kenya | 2990 | 2320 | 6.8% | 8.0% |
| Kuwait | 371 | 248 | 8.6% | 12.6% |
| Kyrgyzstan | 1000 | 777 | 14.2% | 16.8% |
| Laos | 1476 | 1232 | 16.4% | 19.9% |
| Latvia | 1754 | 1265 | 15.8% | 21.8% |
| Lebanon | 1992 | 1535 | 15.7% | 21.4% |
| Lesotho | 114 | 95 | 5.8% | 6.8% |
| Liberia | 166 | 144 | 4.4% | 5.3% |
| Libya | 1099 | 893 | 13.6% | 16.9% |
| Lithuania | 2384 | 1768 | 14.8% | 21.3% |
| Luxembourg | 471 | 290 | 15.8% | 26.7% |
| Madagascar | 1350 | 1031 | 6.5% | 7.3% |
| Malawi | 1142 | 923 | 5.9% | 6.7% |
| Malaysia | 7370 | 5732 | 14.4% | 18.2% |
| Maldives | 70 | 49 | 14.8% | 20.2% |
| Mali | 755 | 608 | 5.1% | 5.8% |
| Malta | 338 | 208 | 12.3% | 20.2% |
| Mauritania | 208 | 168 | 6.5% | 7.6% |
| Mauritius | 293 | 224 | 10.5% | 15.2% |
| Mexico | 17807 | 12856 | 9.0% | 13.5% |
| Moldova | 2408 | 1809 | 18.0% | 22.3% |
| Mongolia | 1176 | 959 | 17.7% | 19.5% |
| Montenegro | 554 | 420 | 22.1% | 27.6% |
| Morocco | 8559 | 7055 | 13.6% | 19.2% |
| Mozambique | 1362 | 1086 | 5.3% | 5.8% |
| Myanmar | 17285 | 14029 | 22.7% | 25.9% |
| Namibia | 268 | 184 | 8.3% | 9.8% |
| Nepal | 4601 | 3516 | 21.1% | 24.1% |
| Netherlands | 22354 | 13155 | 19.2% | 26.5% |
| New Zealand | 3833 | 2401 | 13.9% | 21.6% |
| Nicaragua | 589 | 473 | 7.1% | 9.9% |
| Niger | 464 | 398 | 4.1% | 4.6% |
| Nigeria | 2521 | 1894 | 2.0% | 2.4% |
| North Korea | 14448 | 11706 | 23.6% | 27.5% |
| North Macedonia | 1481 | 1152 | 20.5% | 26.7% |
| Norway | 5455 | 2975 | 14.6% | 22.3% |
| Oman | 241 | 174 | 6.0% | 7.7% |
| Pakistan | 24184 | 17875 | 13.4% | 15.4% |
| Panama | 506 | 360 | 6.2% | 9.6% |
| Papua New Guinea | 1864 | 1221 | 16.2% | 17.6% |
| Paraguay | 1755 | 1194 | 13.1% | 18.4% |
| Peru | 4568 | 3235 | 6.5% | 9.1% |
| Philippines | 32293 | 25643 | 17.2% | 22.7% |
| Poland | 42309 | 30822 | 20.9% | 25.9% |
| Portugal | 10151 | 6728 | 15.2% | 20.1% |
| Qatar | 134 | 89 | 7.8% | 11.4% |
| Republic of the Congo | 94 | 75 | 3.4% | 4.3% |
| Romania | 19434 | 14001 | 19.3% | 25.2% |
| Russia | 110013 | 71889 | 17.9% | 23.2% |
| Rwanda | 568 | 453 | 8.2% | 9.4% |
| Saint Lucia | 37 | 24 | 8.2% | 10.6% |
| Samoa | 62 | 43 | 15.6% | 20.6% |
| Sao Tome and Principe | 13 | 12 | 9.1% | 13.2% |
| Saudi Arabia | 1685 | 1173 | 6.1% | 8.8% |
| Senegal | 594 | 485 | 5.1% | 6.0% |
| Serbia | 9090 | 6626 | 22.4% | 28.0% |
| Sierra Leone | 158 | 134 | 8.2% | 10.0% |
| Singapore | 2972 | 2398 | 12.0% | 18.1% |
| Slovakia | 4792 | 3219 | 16.4% | 21.4% |
| Slovenia | 2190 | 1427 | 16.0% | 20.9% |
| Solomon Islands | 67 | 54 | 9.8% | 14.9% |
| Somalia | 556 | 474 | 5.3% | 6.0% |
| South Africa | 12905 | 9892 | 12.8% | 15.5% |
| South Korea | 37212 | 23127 | 16.0% | 23.8% |
| Spain | 49938 | 29709 | 18.9% | 25.9% |
| Sri Lanka | 4696 | 3205 | 14.3% | 16.9% |
| Sudan | 1473 | 1146 | 5.2% | 6.3% |
| Suriname | 137 | 102 | 12.4% | 16.2% |
| Sweden | 7120 | 4481 | 11.5% | 17.6% |
| Switzerland | 7919 | 4411 | 16.4% | 22.9% |
| Syria | 3018 | 2545 | 14.0% | 18.6% |
| Tajikistan | 442 | 369 | 6.9% | 8.8% |
| Tanzania | 5591 | 4309 | 12.7% | 14.6% |
| Thailand | 32222 | 25469 | 17.9% | 21.6% |
| The Gambia | 107 | 100 | 9.0% | 11.1% |
| Timor-Leste | 125 | 103 | 15.3% | 19.9% |
| Togo | 243 | 187 | 4.6% | 5.3% |
| Trinidad and Tobago | 372 | 269 | 9.5% | 12.1% |
| Tunisia | 3970 | 3087 | 19.8% | 24.8% |
| Turkey | 51065 | 40031 | 22.0% | 31.0% |
| Turkmenistan | 807 | 653 | 12.3% | 14.8% |
| Uganda | 2413 | 1929 | 6.8% | 7.9% |
| Ukraine | 29023 | 20712 | 19.3% | 24.8% |
| United Arab Emirates | 375 | 246 | 6.9% | 10.9% |
| United Kingdom | 77384 | 45293 | 18.5% | 25.1% |
| United States of America | 294628 | 139285 | 16.1% | 23.2% |
| Uruguay | 2851 | 2085 | 18.2% | 23.3% |
| Uzbekistan | 3185 | 2357 | 9.0% | 10.8% |
| Vanuatu | 26 | 25 | 10.5% | 16.5% |
| Venezuela | 7310 | 5139 | 12.5% | 16.5% |
| Vietnam | 36655 | 30153 | 20.5% | 25.2% |
| Yemen | 1580 | 1350 | 9.7% | 11.5% |
| Zambia | 883 | 637 | 5.9% | 6.6% |
| Zimbabwe | 1070 | 864 | 6.2% | 7.4% |

**Data is unavailable for the following countries: Andorra, Antigua and Barbuda, Cook Islands, Dominica, French Guiana, French Polynesia, Grenada, Guadeloupe, Hong Kong, Kiribati, Micronesia (Federated States of), Nauru, New Caledonia, Niue, Reunion, Saint Kitts and Nevis, Saint Vincent and the Grenadines, Seychelles, Swaziland, Taiwan, Tonga, Tuvalu.**

References:

1. Global Cancer Observatory, Ferlay J, Ervik M, Lam F, Laversanne M, Colombet M, et al. Statistics at a Glance, 2022 [Internet]. International Agency for Research on Cancer; 2024. Available from: https://gco.iarc.who.int/media/globocan/factsheets/populations/4-afghanistan-fact-sheet.pdf
2. Spatial, temporal, and demographic patterns in prevalence of smoking tobacco use and attributable disease burden in 204 countries and territories, 1990-2019: a systematic analysis from the Global Burden of Disease Study 2019. Lancet. 2021;397(10292):2337-60.
